# Dung beetle–mammal associations: methods, research trends and future directions

**DOI:** 10.1098/rspb.2018.2002

**Published:** 2019-02-20

**Authors:** Elizabeth H. Raine, Eleanor M. Slade

**Affiliations:** 1 Department of Zoology, University of Oxford, South Parks Road, Oxford OX1 3PS, UK; 2 Lancaster Environment Centre, University of Lancaster, Lancaster LA1 AYQ, UK

**Keywords:** trophic interaction, indicator taxa, scarabaeoidae, conservation, ecosystem functioning, mammals

## Abstract

Dung beetles are increasingly used as a study taxon—both as bioindicators of environmental change, and as a model system for exploring ecosystem functioning. The advantages of this focal taxon approach are many; dung beetles are abundant in a wide range of terrestrial ecosystems, speciose, straightforward to sample, respond to environmental gradients and can be easily manipulated to explore species-functioning relationships. However, there remain large gaps in our understanding of the relationship between dung beetles and the mammals they rely on for dung. Here we review the literature, showing that despite an increase in the study of dung beetles linked to ecosystem functioning and to habitat and land use change, there has been little research into their associations with mammals. We summarize the methods and findings from dung beetle–mammal association studies to date, revealing that although empirical field studies of dung beetles rarely include mammal data, those that do, indicate mammal species presence and composition has a large impact on dung beetle species richness and abundance. We then review the methods used to carry out diet preference and ecosystem functioning studies, finding that despite the assumption that dung beetles are generalist feeders, there are few quantitative studies that directly address this. Together this suggests that conclusions about the effects of habitat change on dung beetles are based on incomplete knowledge. We provide recommendations for future work to identify the importance of considering mammal data for dung beetle distributions, composition and their contributions to ecosystem functioning; a critical step if dung beetles are to be used as a reliable bioindicator taxon.

## 1. Introduction

Indicator species are often used as a more efficient way to assess ecosystem integrity than sampling a large number of taxa [1]. However, for a focal taxon to be used to assess a community habitat, or the effects of environmental change, robust quantitative data and a detailed understanding of its ecology are needed [2]. Dung beetles are an ideal indicator taxon because of their sensitivity to habitat change [3,4], in combination with broad geographical distributions and ease of collection [5–7]. As such, they have been increasingly used as bioindicators to inform conservation management decisions [8–11]. However, the effect of mammal species compositional change on dung beetles and their associated ecosystem functions has been little explored (but see [12–14]).

Dung beetles primarily feed and breed in dung but are also capable of using carrion, rotting fruit, fungi and decaying plant matter [15,16]. As a result, dung beetles contribute to the ecosystem functions of dung removal [17], seed dispersal [18], nutrient cycling [19,20] and reduction of greenhouse gas emissions [21,22]. In this review, we focus on coprophagic dung beetles (Coleoptera: Scarabaeoidea in the families Geotrupidae, Aphodiinae and Scarabaeinae) that feed primarily on dung, and so are expected to have associations with mammals. Dung beetles can be classified into clearly defined functional groups that can be easily manipulated for ecosystem functioning experiments [23], and as a result are increasingly used for understanding trait-functioning associations [24,25]. However, while studies of dung beetles have provided a large body of information on species' distributions and responses to land use change, they rarely shed light on the biotic interactions between dung beetles and mammals [12–14].

Dung beetles are commonly assumed to display generalist feeding and breeding strategies, with many feeding on more than two dung sources [26,27], or using dung from both native and exotic mammal species [28,29]. Mammalian dung varies in nutrient and fibre content among species [30], but also seasonally based on diet [31]. The quality and quantity of the dung provisioned to larvae have been shown to affect the number, size and development of offspring, and to result in resource allocation trade-offs in adult beetles [32,33]. Dung is used in different ways by adults and larvae [30], and selection of dung for breeding may differ to that used for feeding, although the consequences of this for ecosystem functioning has been little explored (but see [14,34,35]). In addition, variation in the digestive system and gut microbia in dung beetles may play a role in determining feeding preferences [36–38], and while dung volatiles are thought to be key to determining the attractiveness of dung to dung beetles [39,40], how they relate to dung beetle resource use is still not well understood [30,41].

Mammals are also often used as an indicator or flagship taxon [42,43], and there has been extensive research concerning mammal species responses to disturbance (e.g. [44,45]), and species’ associations to habitat types [46]. However, in contrast to dung beetles, mammals are notably harder to survey, requiring more time, effort and at a greater cost [47]. Yet, despite their close ecological association, and despite being two of the best studied vertebrate and invertebrate taxa individually, dung beetles and mammals are rarely studied in combination [48] (but see ‘Avenues for future work’ below for the potential of new molecular methods).

We conducted a systematic review of studies documenting the associations between dung beetles and mammals to address the following questions: (i) what have been the research trends in the study of dung beetle ecology? (ii) what methodological approaches are used to study dung beetle–mammal associations, and dung beetle–dung associations? and (iii) is diet preference and association with mammals accounted for in studies of dung beetle ecosystem functioning? We then highlight the knowledge gaps and give recommendations for how dung beetle–mammal interactions can be incorporated into future work on dung beetle ecology.

## 2. Material and methods

We carried out a review of the literature to identify trends in the study of dung beetle ecology using Web of Science (as of 18 December 2018). A 1990 start year was chosen to represent a shift in focus towards human land use change and ecosystem functioning in ecology [49]. All searches used the keyword topic (‘dung beetle’ OR scarabaeinae OR geotrupidae OR aphodiinae). We identified literature considering dung beetle–mammal associations using the search term (mammal* OR preference* OR diet*). Additional papers were identified by following publications cited in these articles. The papers were then reviewed to identify the approach to classifying the dung beetle–mammal associations and interactions (box 1). We distinguished between studies that tested interactions directly through dung beetle dietary preference experiments using different dung types (box 1*a*,*b*), and those that used mammal and dung beetle co-occurrence data to indirectly infer associations between the two (box 1*c*,*d*). We then systematically reviewed the approach taken to classifying the dung beetle–mammal associations in the dung beetle–mammal co-occurrence studies. We recorded three aspects of the studies: (i) how the mammal composition was classified; (ii) how the analysis of the association between dung beetles and mammals was carried out; and (iii) the direction of the effect of the mammal communities on the dung beetle population metrics. For diet preference studies, only those that used at least two dung types were included. For these studies, we identified the location, as well as the dung types and dung volumes used, and the experimental design employed in each of the studies.

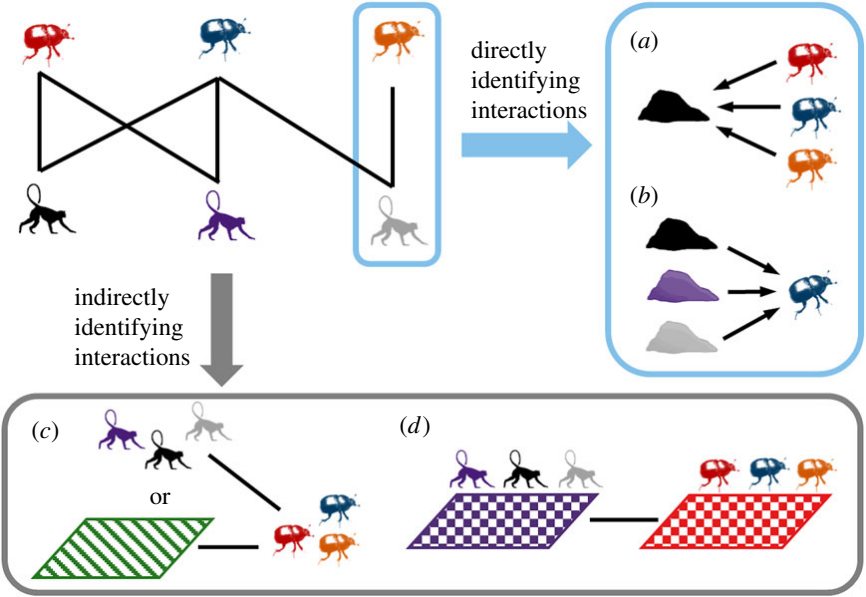
Box 1. The different approaches to studying dung beetle–mammal associations. The dung beetle–mammal interaction network can be estimated directly (*a,b*) or indirectly (*c,d*). (*a*) Directly identifying the dung beetle species composition attracted to a dung type; (*b*) identifying dung beetle species resource use breadth and dietary preference by identifying attraction to multiple dung types; (*c*) inferring population level associations between dung beetles and mammals either via pooled mammal composition data or via a proxy for mammal composition such environmental condition; and (*d*) inferring species-level interactions between dung beetles and mammals via associations between species that co-occur.

To compare the number of dung beetle–mammal association studies with the number of other studies to provide a minimum indicator of the amount of ecological survey work documenting the study of dung beetles, two additional searches were undertaken. This used the keyword search topic: (habitat* OR environment*) for dung beetle–habitat associations, and: (ecosystem* service* OR ecosystem* function*) for ecosystem function studies. We excluded studies exploring the effects of pesticide and insecticide application on ecosystem functions as this was beyond the scope of this review. This literature search was not exhaustive, as papers that did not refer specifically to these search terms were not further identified. Studies concerning dung beetle ecosystem functioning were assessed for the extent to which they considered feeding variation among dung beetle species. For each study, we recorded location, the number of dung types used, dung volumes and ecosystem function(s) measured.

## 3. Results

### (a) Trends in dung beetle ecological research

**Figure 1.**
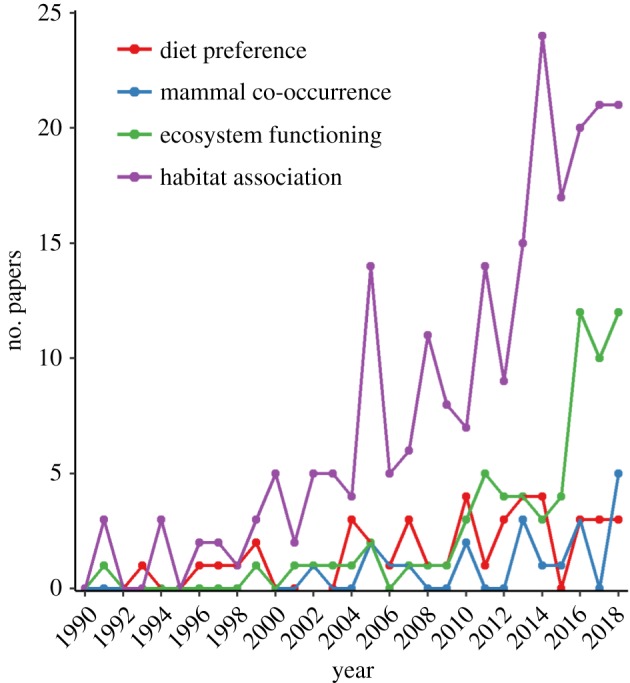
Journal articles published on dung beetle ecology from 1990 to 2018, details of papers given in the electronic supplementary material, appendix A.

**Figure 2.**
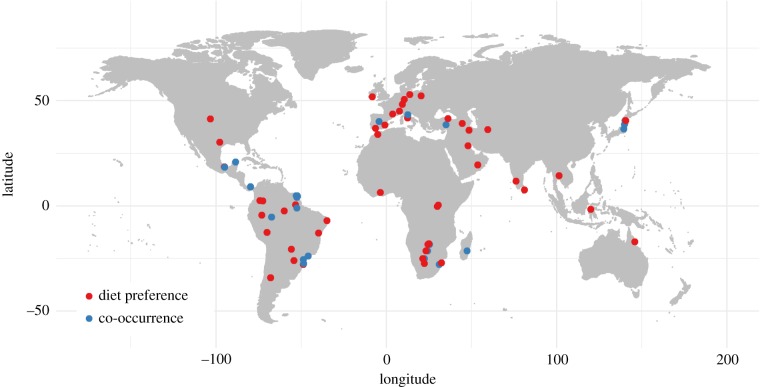
Study locations for papers addressing dung beetle–mammal association studies, separated into mammal co-occurrence and diet preference studies (field-based—see below) (1990–2018). Where the study conducted sampling at disparate locations, multiple points are included.

The literature review yielded a total of 359 papers from all three searches. Studies of dung beetle–mammal associations accounted for 65 papers, 68 papers focused on dung beetle–ecosystem functioning research and 226 studies consisted of empirical field studies of dung beetle–habitat associations (see the electronic supplementary material, appendix A). Forty-four of the dung beetle–mammal association studies addressed direct interactions and dung beetle dietary preference, and 21 considered the indirect effect of mammal presence on dung beetle populations or community composition. These two categories were explored separately. There has been an increase in publications on ecosystem functions and habitat associations using dung beetles since 1990 (figure 1). By contrast, studies of dung beetle–mammal associations, both diet preference and species co-occurrence studies, have remained low throughout this period, with studies largely focused in Europe and South America, and with relatively few studies in Asia, Australasia, Africa and North America (figure 2).

### (b) Dung beetle–mammal association studies: diet preference and direct interactions

Of the 44 diet preference studies, 89% were field experiments and 11% laboratory experiments (box 1*a*,*b*). The most commonly used study design for field surveys included only two dung types and distance between traps varied from 1 to 100 m (mode = 50 m) (electronic supplementary material, table S2, figure S1). Experimental methods also varied, with studies either using pitfall traps, directly collecting dung beetles from dung, or using burial intercept traps (see [35]). Cattle dung was the most commonly used dung type. All but one laboratory study [40] assessed diet preference for just one dung beetle species. Across the 68 papers that studied ecosystem functioning, the majority used only one dung type, again with cattle dung the most frequently used bait (electronic supplementary material, figure S1, table S3). Most studies considered only one ecosystem function, dung removal, but other studies also addressed seed removal and dispersal, nutrient cycling and plant growth. The dung volume used in ecosystem functioning studies varied greatly and larger volumes were used in comparison to dietary preference studies (electronic supplementary material, figure S1).

### (c) Dung beetle–mammal association studies: co-occurrence data and indirect interactions

There were 21 studies that assessed dung beetle–mammal associations through their co-occurrence (box 1*c*,*d*) (electronic supplementary material, table S1). Two studies used co-occurrence data to estimate species-level interactions between dung beetles and mammals [13,50] (box 1*d*). The other studies assessed population-level associations using a range of approaches to identify mammal composition, including proxies, individual focal mammal species, grazing intensity or estimates of mammal composition based on species richness and raw abundance (box 1*c*). Thirty-eight per cent of the studies included information on small mammals in their estimates of mammal species composition. Three studies estimated dung beetle occurrence using a range of different dung types [14,51,52]. In each of the studies, we identified the effect a decline in mammal abundance or richness had on the dung beetle population. All studies showed at least one positive association between dung beetle species richness and abundance and the mammal metric used, indicating that a reduction in the mammal community had a negative effect on the dung beetle community. Dung beetle community composition was significantly different between areas of varying mammal composition for all but two studies.

## 4. Discussion

There has been a rapid increase in the study of coprophagous dung beetles over the last 29 years, yet despite their reliance on mammal dung [48], the understanding of dung beetle–mammal associations is still limited. Increasing data on dung beetle associations with the environment and their functional contributions to ecosystems has not been mirrored by an advancement of the mechanistic understanding of how dung beetles use dung for feeding and breeding, the cascading consequences of defaunation for dung beetle populations, and the ecosystem functions they provide. This lack of research is apparent across both tropical and temperate regions.

### (a) Dung beetle–mammal associations

We found that studies on the effects of mammals on dung beetle community composition, species richness and abundance have largely been neglected, despite calls to increase the inclusion of mammal data in dung beetle studies [48]. Although the dung beetle–mammal co-occurrence studies are revealing about the importance of individual mammal species [52,53] and the effects of grazing intensity on dung beetle populations [54,55], the majority were based on proxies or qualitative estimates of mammal composition (box 1*c*,*d*). Several studies identified the importance of large bodied mammals for dung beetle species composition [52,56], but only a third of studies accounted for small bodied mammals in their assessment of faunal composition. Overall, studies reported consistent trends towards co-declines in dung beetles and mammals [12,57,58]. This suggests that changes in mammal species composition, such as those occurring as a result of habitat disturbance, are likely to have significant impacts on dung beetle communities, and associated ecosystem functioning (see [48] for review).

### (b) Dung beetle diet preference and identifying direct interactions

It is commonly assumed that dung beetles show broad dietary widths [26,59], and a recent meta-analysis suggests generalist feeding preferences in dung beetles across latitudinal gradients [27]. However, the range of experimental methods used in the majority of the studies reviewed here does not provide conclusive evidence to support this. Many studies compared the attractiveness of just two dung types [60–62], or analysed dung beetle species composition attracted to single dung types (box 1*a*), rather than individual dung beetle species feeding breadth (box 1*b*) [63–66]. Several studies show that dung beetle species can vary in their attraction to the dung of different mammal trophic groups [67,68], and particular mammal species [69–71]. Equally experimental choice trials in the laboratory have shown variation in dung beetle species resource use [37,72]. Although more time consuming, feeding and breeding choice experiments in the field are key to enabling mechanistic questions surrounding attractiveness of dung to dung beetles to be addressed, such as identifying the association between dung type used for brood provisioning and adult body size [31,73].

In the past 20 years, the study of biodiversity–ecosystem functioning relationships has grown markedly and dung beetles are increasingly used as a focal taxon for such studies [74]. In this review, 60% of ecosystem functioning studies used only one dung type, and over 50% of the time this was domesticated animal dung. As a consequence, variation in the choice between feeding and breeding—two functionally very different interactions—is still unknown. Although livestock dung is important for dung beetle populations globally, especially in agro-ecosystems, it can provide a limited snapshot of the extent of ecosystem functions provided by dung beetles, especially those not performed by domestic animals, such as seed dispersal [75,76], and their important role in non-pasture ecosystems, such as tropical forests [17,77].

### (c) Regional variation

Studies exploring dung beetle–mammal associations were concentrated in the Neotropics and Europe, with few studies carried out elsewhere. Variation among biogeographical regions in mammal fauna is likely to have impacted the evolution of dung beetle–mammal associations [78]. In the Neotropics and temperate regions, such as Europe and North America, mammalian biomass is low owing to high rates of mammal extinction in the Pleistocene [72]. This may have resulted in dung beetles switching to non-mammalian dung food sources such as fruit, fungi, carrion, or plant detritus, or developing greater plasticity in their diets [72,79–81]. By contrast, the mammal fauna in Africa is dominated by large herbivorous species and there appears to be a higher number of beetles specialized in coprophagy, with fruit and carrion feeding recorded less frequently [15,79,82]. In addition, regional differences in environmental conditions, such as the dominance of savannahs versus humid forests effect the abundance of dung beetles and attraction to different food sources [83].

### (d) Avenues for future work

**Figure 3.**
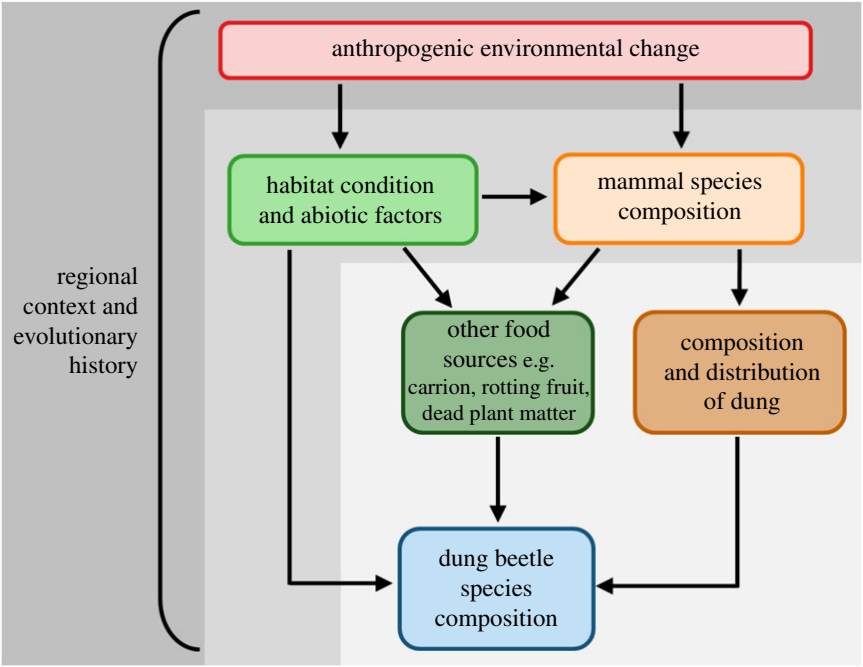
Conceptual diagram of the processes by which environmental change can affect coprophagous dung beetle composition and ecosystem functioning, with avenues for future work represented. Lightest grey: (i) identifying direct interactions between dung beetles and their resources (mammal dung and others); medium grey: (ii) decoupling the effects of biotic factors (availability of resources) from abiotic factors and habitat change on dung beetle populations; dark grey: (iii) identifying dietary switching and plasticity in dung beetles in response to defaunation, within the evolutionary context of different regions.

Here we identify areas to improve the understanding of dung beetle–mammal associations within the context of environmental change and advancing the understanding of ecosystem functions provided by dung beetles (figure 3).

#### (i) Identifying dung beetle resource use

*Standardized survey methods.* Our review highlights the lack of consistent and comparable methods in the experimental design of both diet preference and ecosystem functioning studies, making a synthesis of results challenging. The composition of dung beetles captured in traps is affected by dung volume [84] and trap spacing [85,86], and trapping methods to detect the difference between the choice of dung for feeding and breeding have only recently been developed [34,35]. In addition, the efficacy of different trapping methods and how dung attractiveness to dung beetle species varies across different habitats [87,88] and between life cycle stages [14] is not well understood. Future work is needed to identify standardized experimental designs that strive to minimize the effects of these possible variables. Moreover, the prevalence of studies using only one or two dung types prevents the direct interactions between mammals and dung beetles from being described. We call for standardization of survey methods, and incorporation of surveying with realistic dung sizes of multiple native species. This will enable more accurate predictions of how environmental change and, specifically defaunation will affect these interactions, and the consequences for ecosystem functions and services.

*The importance of natural history, taxonomy and museum collections.* Establishing species-level interactions is crucial to answering questions about whole interaction networks and the impacts of environmental change at larger scales [89,90]. However, this relies on an understanding of the taxonomy and natural history of the species involved; knowledge which is currently lacking for many dung beetle species, particularly in the tropics. Recently, there has been a renewed interest in the importance of natural history, and concern over the decline of taxonomy, with calls for increased funding and research for these areas [90–92]. As well as increased observational and natural history studies in the field, using the large amounts of data held in museum collections is crucial [89], and unpublished sources, species notes and observations provide a key contribution to our understanding of dung beetle ecology [15].

*Using new molecular methods to understand direct interactions.* Advances in DNA barcoding mean it is now possible to identify vertebrate genetic material from invertebrates that feed on them [93,94]. DNA in dung beetle gut contents has been used to successfully identify the mammal species dung they fed on in pilot studies [95–97]. Such studies have the potential to be an important addition to mammal survey data, particularly in detecting rare or cryptic species. The attractiveness of these new molecular methods for studying dung beetle mammal interactions is that they have the potential to sidestep the issues affecting ecological sampling (e.g. the size and dung type of the bait used in the trap) and allow direct identification of species interactions under natural conditions.

#### (ii) Identifying drivers of dung beetle responses to environmental change

*Incorporating mammals into dung beetle studies.* To understand the responses of dung beetles to disturbance, it is necessary to consider both biotic and abiotic conditions. There has been much focus recently on the declines of insects owing to habitat loss [98] but the importance of abiotic and biotic factors in driving this response is unknown for most species. Thus, decoupling these drivers is crucial to inform conservation planning for insect populations globally. In this context, the lack of studies including mammals as a biotic factor in analyses of dung beetle responses to environmental change neglects an important factor influencing their distribution and responses. However, disentangling the effects of mammal species presence, abundance and richness on dung beetle species composition from abiotic factors is still a challenge which can only be addressed by accounting for both taxa in population level assessments. Often expensive field equipment employed in the field over long time periods is required to gather the high-resolution mammal data required for this kind of analysis (but see above for molecular advances). This work can be furthered to understand how the reintroduction of mammal species can affect dung beetle recovery in restoration projects [8,52].

*Identifying important functional traits.* Dung beetle responses to environmental change can also be considered in terms of shared functional traits among species that both shape their use of the resource (effect traits) and how they respond to their environment and changes in it (response traits) [25]. Although several dung beetle functional traits are known to be important in influencing how species respond to environmental change [77], the traits relating to resource use and dietary preference (such as olfaction [41,99], flight capacity [100], digestion [101] and searching behaviour [102]) are not well understood.

*Modelling interactions from co-occurrence data.* Identifying ecological interactions from community patterns in species occurrence from spatial data is commonplace [103], where non-random species occurrence patterns are used to infer interspecific interactions [104,105]. Advances in the use of species distribution modelling have recently been applied to dung beetle–mammal co-occurrence data to reveal interactions between the two trophic levels, and also identify the effect of ecological processes such as dispersal, in addition to abiotic factors, on dung beetle species distributions [106–108].

#### (iii) Identifying dietary switching and plasticity in dung beetles

*Dietary switching and plasticity in resource use.* The relative importance of alternative food sources such as rotting fruit, fungi and carrion, and plant detritus impact the extent of interactions between dung beetles and mammals and should be included in dung beetle feeding studies. Research addressing dietary plasticity has mostly been conducted in the Neotropics (see [15] for review), and the importance of these resources as alternatives in dung beetle diets in other regions is largely unknown. Pleistocene extinctions of the large mega-fauna in the Neotropics and Europe [72,109], and historical mammal species introductions have resulted in dung use switching in native adult dung beetle species in these regions, either to alternative dung sources [110,111] or to frugivory [112]. In addition, flexibility in resource choice for breeding, and the impact that this has on larval development and survival is not well understood [15,41]. A quantitative network approach may be a valuable way to assess resource use and flexibility in dung beetles, especially under changing environmental conditions [14].

*Regional trends in research and evolutionary context.* The current abiotic and biotic conditions in combination with historical context affects the ability of dung beetles to adapt to new environmental conditions and the introduction of exotic mammal dung [13,77,83,113]. However, the extent to which each of these factors contributes to the ability of dung beetles to adapt to changing conditions and the extent to which resource switching and diet plasticity exist is still vastly understudied [79]. Few studies fully address the range and availability of food sources available in a study area, and in particular, the dung of small mammals is rarely included. More studies are needed outside of the Neotropics to test whether biogeographical differences in diet flexibility are a result of differences in resource availability and adaptation in dung beetles among regions, or simply a sampling effect from the limited research conducted to date [15,72].

### (e) Synthesis

Although there has been an increase in dung beetle research related to their response to disturbance and their importance for ecosystem functioning, further work is now needed to put this into context with regard to their resource use, interactions with mammals and evolutionary history. The use of dung beetles as bioindicators is currently constrained by a lack of understanding of the associations between dung beetles and mammals; a necessary pre-requisite if data are to be scaled up to give a broader understanding of the extent of functioning provided by dung beetles within whole ecosystems, and their response to environmental change.

## Supplementary Material

Appendix A, Supplementary Tables S1, S2, S3

## Data accessibility

This article has no additional data.
