# Supplementary material for: Dung beetle–mammal associations: methods, research trends and future directions
Source: Proc Biol Sci. 2019 Feb 20;286(1897):20182002. doi: 10.1098/rspb.2018.2002 (PMC6408906; doi:10.1098/rspb.2018.2002)
Supplement: Appendix A, Supplementary Tables S1, S2, S3 [file rspb20182002supp1.docx]

**Supplementary material**

**Appendix A.** Supplementary data. List of references on dung beetle ecology research from the bibliographic search from 1991- 2017

**Diet preference studies:**

1. Al-Houty W, Al-Musalam F. 1997 Dung preference of the dung beetleScarabaeus cristatusFab (Coleoptera-Scarabaeidae) from Kuwait. *J. Arid Environ.* 35, 511–516. (doi:10.1006/jare.1996.0179)
2. Amézquita S, Favila ME. 2010 Removal rates of native and exotic dung by dung beetles (Scarabaeidae: Scarabaeinae) in a fragmented tropical rain forest. *Environ. Entomol.* 39, 328–336. (doi:10.1603/EN09182)
3. Barbero E, Palestrini C, Rolando A. 1999 Dung beetle conservation: effects of habitat and resource selection (Coleoptera: Scarabaeoidea). *J. Insect Conserv.* (doi: 10.1023/A:1009609826831)
4. Bogoni JA, Hernández MIM. 2014 Attractiveness of Native Mammal’s Feces of Different Trophic Guilds to Dung Beetles (Coleoptera: Scarabaeinae). *J. Insect Sci.* 14, 1–7. (doi:10.1093/jisesa/ieu161)
5. Carpaneto GM, Mazziotta A, Ieradi M. 2010 Use of Habitat Resources by Scarab Dung Beetles in an Savanna. *Environ. Entomol.* 39, 1756–1764. (doi:10.1603/EN09249)
6. Carpaneto GM, Mazziotta A, Piattella E. 2005 Changes in food resources and conservation of scarab beetles: from sheep to dog dung in a green urban area of Rome (Coleoptera, Scarabaeoidea). *Biol. Conserv.* 123, 547–556. (doi:10.1016/j.biocon.2004.12.007)
7. Correa CMA, Puker A, Korasaki V, Ferreira KR, Abot AR. 2016 Attractiveness of baits to dung beetles in Brazilian savanna and exotic pasturelands. *Entomol. Sci.* 19, 112–123. (doi:10.1111/ens.12169)
8. Davis ALV, Scholtz CH, Kryger U, Deschodt CM, Strümpher WP. 2010 Dung beetle assemblage structure in Tswalu Kalahari Reserve: responses to a mosaic of landscape types, vegetation communities, and dung types. *Environ. Entomol.* 39, 811–820. (doi:10.1603/EN09256)
9. Dormont L, Rapior S, McKey D, Lumaret J. 2007 Influence of dung volatiles on the process of resource selection by coprophagous beetles. *Chemoecology* 17, 23–30. (doi: 10.1007/s00049-006-0355-7)
10. Dormont L, Epinat G, Lumaret J-P. 2004 Trophic Preferences Mediated by Olfactory Cues in Dung Beetles Colonizing Cattle and Horse Dung. *Environ. Entomol.* 33, 370–377. (doi:10.1603/0046-225X-33.2.370)
11. Edwards PB. 1991 Seasonal Variation in the Dung of African Grazing Mammals, and its Consequences for Coprophagous Insects. *Funct. Ecol.* 5, 617. (doi:10.2307/2389480)
12. Enari H, Koike S, Sakamaki H. 2013 Influences of different large mammalian fauna on dung beetle diversity in beech forests. *J. Insect Sci.* 13, 54. (doi:10.1673/031.013.5401)
13. Enari H, Koike S, Sakamaki-Enari H. 2016 Ecological implications of mammal feces buried in snow through dung beetle activities. *J. For. Res.* 21, 92–98. (doi:10.1007/s10310-015-0516-z)
14. Errouissi F, Haloti S, Jay-robert P, Janati-idrissi A, Lumaret J-P. 2004 Effects of the Attractiveness for Dung Beetles of Dung Pat Origin and Size Along a Climatic Gradient. *Environ. Entomol.* 33, 45–53. (doi:10.1603/0046-225X-33.1.45)
15. Estrada A, Halffter G, Coates-Estrada R, Meritt DA. 1993 Dung Beetles Attracted to Mammalian Herbivore (*Alouatta palliata*) and Omnivore (*Nasua narica*) Dung in the Tropical Rain Forest of Los Tuxtlas, Mexico. 9, 45–54. (doi:10.1017/S0266467400006933)
16. Filgueiras BKC, Liberal CN, Aguiar CDM, Hernández MIM, Iannuzzi L. 2009 Attractivity of omnivore, carnivore and herbivore mammalian dung to Scarabaeinae (Coleoptera, Scarabaeidae) in a tropical Atlantic rainforest remnant. *Rev. Bras. Entomol.* 53, 422–427. (doi: 10.1590/S0085-56262009000300017)
17. Finn J, Giller P. 2002 Experimental investigations of colonisation by north temperate dung beetles of different types of domestic herbivore dung. *Appl. Soil Ecol.* (doi: 10.1016/S0929-1393(02)00011-2)
18. Frank K, Brückner A, Hilpert A, Heethoff M, Blüthgen N. 2017 Nutrient quality of vertebrate dung as a diet for dung beetles. *Sci. Rep.* 7, 1–12. (doi:10.1038/s41598-017-12265-y)
19. Frank K, Hülsmann M, Assmann T, Schmitt T, Blüthgen N. 2017 Land use affects dung beetle communities and their ecosystem service in forests and grasslands. *Agric. Ecosyst. Environ.* 243, 114–122. (doi:10.1016/j.agee.2017.04.010)
20. Galante E, Cartagena MC. 1999 Comparison of Mediterranean Dung Beetles (Coleoptera: Scarabaeoidea) in Cattle and Rabbit Dung. *Environ. Entomol.* 28, 420–424. (doi:10.1093/ee/28.3.420)
21. Gittings T, Giller P. 1998 Resource quality and the colonisation and succession of coprophagous dung beetles. *Ecography (Cop.).* 21, 581-592. (doi: 10.1111/j.1600-0587.1998.tb00550.x)
22. Hewavithana DK, Wijesinghe MR, Dangalle CD, Dharmarathne HASG. 2016 Habitat and dung preferences of scarab beetles of the subfamily Scarabaeinae: a case study in a tropical monsoon forest in Sri Lanka. *Int. J. Trop. Insect Sci.* 36, 97–105. (doi:10.1017/S1742758416000023)
23. Jones AG, Forgie SA, Scott DJ, Beggs JR. 2012 Generalist dung attraction response in a New Zealand dung beetle that evolved with an absence of mammalian herbivores. *Ecol. Entomol.* 37, 124–133. (doi:10.1111/j.1365-2311.2012.01344.x)
24. Larsen TH, Lopera A, Forsyth A. 2006 Extreme Trophic and Habitat Specialization by Peruvian Dung Beetles (Coleoptera: Scarabaeidae: Scarabaeinae). *Coleopt. Bull.* 60, 315–324. (doi:10.1649/0010-065X(2006)60[315:ETAHSB]2.0.CO;2)
25. Marsh CJ, Louzada J, Beiroz W, Ewers RM. 2013 Optimising bait for pitfall trapping of Amazonian dung beetles (Coleoptera: Scarabaeinae). *PLoS One* 8, e73147. (doi:10.1371/journal.pone.0073147)
26. Martín-Piera F, Lobo J. 1996 A comparative discussion of trophic preferences in dung beetle communities. *Misc. Zool.* 19, 13--31.
27. Medina A, Lopes P. 2014 Resource Utilization and Temporal Segregation of Scarabaeinae (Coleoptera, Scarabaeidae) Community in a Caatinga Fragment. *Neotrop. Entomol.* (doi:10.1007/s13744-014-0198-9)
28. Mroczyński R, Komosiński K. 2014 Differences between beetle communities colonizing cattle and horse dung. *Eur. J. Entomol.* 111, 349–355. (doi:10.14411/eje.2014.050)
29. Noriega JA. 2012 Dung Beetles (Coleoptera: Scarabaeinae) Attracted to *Lagothrix lagotricha* (Humboldt) and *Alouatta seniculus* (Linnaeus) (Primates: Atelidae) Dung in a Colombian Amazon Forest. *Psyche A J. Entomol.* 2012, 1–6. (doi:10.1155/2012/437589)
30. Plewińska B. 2007 The effect of food odour on food preference, activity and density of dung beetle *Geotrupes stercorosus* (Scriba, 1791) in a mixed coniferous forest. *Polish J. Ecol.* 55, 495–509.
31. Puker A, Correa CM a, Korasaki V, Ferreira KR, Oliveira NG. 2013 Dung beetles (Coleoptera: Scarabaeidae) attracted to dung of the largest herbivorous rodent on earth: a comparison with human feces. *Environ. Entomol.* 42, 1218–25. (doi:10.1603/EN13100)
32. Santos-Heredia C, Andresen E, Stevenson P. 2011 Secondary seed dispersal by dung beetles in an Amazonian forest fragment of Colombia: Influence of dung type and edge effect. *Integr. Zool.* 6, 399–408. (doi:10.1111/j.1749-4877.2011.00261.x)
33. Shahabuddin, Hidayat P, Manuwoto S, Noerdjito WA, Tscharntke T, Schulze CH. 2010 Diversity and body size of dung beetles attracted to different dung types along a tropical land-use gradient in Sulawesi, Indonesia. *J. Trop. Ecol.* 26, 53–65. (doi:10.1017/S0266467409990423)
34. Siddall E. 2004 A study of edge effects and dung preference in dung beetles in Kibale. *Masters Diss.*
35. Stavert JR, Gaskett AC, Scott DJ, Beggs JR. 2014 Dung beetles in an avian-dominated island ecosystem: feeding and trophic ecology. *Oecologia* 176, 259–271. (doi:10.1007/s00442-014-3001-z)
36. Tshikae BP, Davis ALV, Scholtz CH. 2013 Dung beetle assemblage structure across the aridity and trophic resource gradient of the Botswana Kalahari: Patterns and drivers at regional and local scales. *J. Insect Conserv.* 17, 623–636. (doi:10.1007/s10841-013-9547-y)
37. Tshikae BP, Davis ALV, Scholtz CH. 2008 Trophic associations of a dung beetle assemblage (Scarabaeidae: Scarabaeinae) in a woodland savanna of Botswana. *Environ. Entomol.* 37, 431–441. (doi:10.1603/0046-225X(2008)37[431:TAOADB]2.0.CO;2)
38. Vernes K, Pope LC, Hill CJ, Bärlocher F. 2005 Seasonally, dung specificity and competition in dung beetle assemblages in the Australian Wet Tropics, north-eastern Australia. *J. Trop. Ecol.* 21, 1–8. (doi:10.1017/S026646740400224X)
39. Vinod K V, Sabu TK. 2007 Species composition and community structure of dung beetles attracted to dung of gaur and elephant in the moist forests of South Western Ghats. *J. Insect Sci.* 7, 1–14. (doi:10.1673/031.007.5601)
40. Whipple SD, Hoback WW. 2012 A Comparison of Dung Beetle (Coleoptera: Scarabaeidae) Attraction to Native and Exotic Mammal Dung. *Environ. Entomol.* 41, 238–244. (doi:10.1603/EN11285)
41. Wurmitzer C, Blüthgen N, Krell FT, Maldonado B, Ocampo F, Müller JK, Schmitt T. 2017 Attraction of dung beetles to herbivore dung and synthetic compounds in a comparative field study. *Chemoecology* 27, 75–84. (doi:10.1007/s00049-017-0232-6)
42. Giménez Gómez VC, Verdú JR, Gómez-Cifuentes A, Vaz-de-Mello FZ, Zurita GA. 2018 Influence of land use on the trophic niche overlap of dung beetles in the semideciduous Atlantic forest of Argentina. *Insect Conserv. Divers.* **11**, 554–564. (doi:10.1111/icad.12299)
43. Sites RW, Lago P, Gale GA. 2018 Associations of scarab beetles (Insecta: Coleoptera: Scarabaeidae) with dung of four species of mammals in Khao Yai National Park, Thailand. *Raffles Bull. Zool.* 66, 87–95.
44. Frank K, Brückner A, Blüthgen N, Schmitt T. 2018 In search of cues: dung beetle attraction and the significance of volatile composition of dung. *Chemoecology* 28, 145–152. (doi:10.1007/s00049-018-0266-4)

**Habitat association studies:**

1. Cajaiba RL, Périco E, da Silva WB, Leote P, Santos M. 2018 Are Small Dung Beetles (Aphodiinae) useful for monitoring neotropical forests’ ecological status? Lessons from a preliminary case study in the Brazilian Amazon. *For. Ecol. Manage.* **429**, 115–123. (doi:10.1016/j.foreco.2018.07.005)

2. Pinto Leite CM, Mariano-Neto E, Bernardo da Rocha PL. 2018 Biodiversity thresholds in invertebrate communities: The responses of dung beetle subgroups to forest loss. *PLoS One* **13**, 1–18. (doi:10.1371/journal.pone.0201368)

3. da Silva PG, Hernández MIM, Heino J. 2018 Disentangling the correlates of species and site contributions to beta diversity in dung beetle assemblages. *Divers. Distrib.* **24**, 1674–1686. (doi:10.1111/ddi.12785)

4. Sánchez Hernández G, Gómez B, Delgado L, Rodríguez-López ME, Chamé-Vázquez ER. 2018 Diversidad de escarabajos copronecrófagos (Coleoptera: Scarabaeidae: Scarabaeinae) en la Reserva de la Biosfera Selva El Ocote, Chiapas, México. *Caldasia* **40**, 144–160. (doi:10.15446/caldasia.v40n1.68602)

5. Ospina-Garcés SM, Escobar F, Baena ML, Davis ALV, Scholtz CH. 2018 Do dung beetles show interrelated evolutionary trends in wing morphology, flight biomechanics and habitat preference? *Evol. Ecol.* **32**, 663–682. (doi:10.1007/s10682-018-9958-z)

6. LaScaleia MC, Reynolds C, Magagula CN, Roets F, McCleery RA. 2018 Dung beetle richness decreases with increasing landscape structural heterogeneity in an African savanna-agricultural mosaic. *Insect Conserv. Divers.* **11**, 396–406. (doi:10.1111/icad.12290)

7. Sullivan CD, Slade EM, Bai M, Shi K, Riordan P. 2018 Evidence of forest restoration success and the conservation value of community-owned forests in Southwest China using dung beetles as indicators. *PLoS One* **13**, e0204764. (doi:10.1371/journal.pone.0204764)

8. Arriaga-Jiménez A, Rös M, Halffter G. 2018 High variability of dung beetle diversity patterns at four mountains of the Trans-Mexican Volcanic Belt. *PeerJ* **6**, e4468. (doi:10.7717/peerj.4468)

9. Reynolds C *et al.* 2018 Inconsistent effects of landscape heterogeneity and land-use on animal diversity in an agricultural mosaic: a multi-scale and multi-taxon investigation. *Landsc. Ecol.* **33**, 241–255. (doi:10.1007/s10980-017-0595-7)

10. Marsh CJ, Feitosa RM, Louzada J, Ewers RM. 2018 Is β-diversity of Amazonian ant and dung beetles communities elevated at rainforest edges? *J. Biogeogr.* **45**, 1966–1979. (doi:10.1111/jbi.13357)

11. Portela Salomão R, González-Tokman D, Dáttilo W, López-Acosta JC, Favila ME. 2018 Landscape structure and composition define the body condition of dung beetles (Coleoptera: Scarabaeinae) in a fragmented tropical rainforest. *Ecol. Indic.* **88**, 144–151. (doi:10.1016/j.ecolind.2018.01.033)

12. Simons P, Molina M, Hagadorn MA, Price DL. 2018 Monitoring of Dung Beetle (Scarabaeidae and Geotrupidae) Activity Along Maryland’s Coastal Plain. *Northeast. Nat.* **25**, 87–100. (doi:10.1656/045.025.0108)

13. Correa CMA, Braga RF, Puker A, Abot AR, Korasaki V. 2018 Optimising Methods for Dung Beetle (Coleoptera: Scarabaeidae) Sampling in Brazilian Pastures. *Environ. Entomol.* **47**, 48–54. (doi:10.1093/ee/nvx191)

14. Giménez Gómez VC, Verdú JR, Guerra Alonso CB, Zurita GA. 2018 Relationship between land uses and diversity of dung beetles (Coleoptera: Scarabaeinae) in the southern Atlantic forest of Argentina: which are the key factors? *Biodivers. Conserv.* **27**, 3201–3213. (doi:10.1007/s10531-018-1597-8)

15. deCastro-Arrazola I, Hortal J, Moretti M, Sánchez-Piñero F. 2018 Spatial and temporal variations of aridity shape dung beetle assemblages towards the Sahara desert. *PeerJ* **6**, e5210. (doi:10.7717/peerj.5210)

16. Raine EH, Gray CL, Mann DJ, Slade EM. 2018 Tropical dung beetle morphological traits predict functional traits and show intraspecific differences across land uses. *Ecol. Evol.* **8**, 8686–8696. (doi:10.1002/ece3.4218)

17. da Silva PG, Lobo JM, Hensen MC, Vaz-de-Mello FZ, Hernández MIM. 2018 Turnover and nestedness in subtropical dung beetle assemblages along an elevational gradient. *Divers. Distrib.* **24**, 1277–1290. (doi:10.1111/ddi.12763)

18. González-Tokman D, Cultid-Medina C, Díaz A, Escobar F, Ocampo-Palacio L, Martínez-Garza C. 2018 Success or failure: the role of ecological restoration on the recovery of dung beetle diversity and function in a tropical rainforest. *Rev. Mex. Biodivers.* **89**, 232–242. (doi:10.22201/ib.20078706e.2018.1.2132)

19. Mongyeh ET, Philips TK, Kimbi HK, Fokam EB. 2018 Elevational and possible bushmeat exploitation effects on dung beetle (Scarabaeidae: Scarabaeinae) communities on Mount Cameroon, West Central Africa. *Environ. Entomol.* **47**, 1072–1082. (doi:10.1093/ee/nvy112)

20. Jugovic J, Koprivnikar N, Koren T. 2018 The role of semi-natural grasslands and livestock in sustaining dung beetle communities (Coleoptera, Scarabaeoidea) in sub-Mediterranean areas of Slovenia. *Anim. Biodivers. Conserv.* **41**, 321–332.

21. Yanes-Gómez G, Tamariz-Flores JV, Silva-Gómez SE, Espejel BO-, Castelán-Vega R del C. 2018 Scarabaeinae Species in the Atoyac-Tehuitzingo Basin at Puebla, Mexico: Diversity and Use as Bioindicators. *Southwest. Entomol.* **43**, 939–946. (doi:10.3958/059.043.0411)

22. Silva RJ, Pelissari TD, Krinski D, Canale G, Vaz-de-Mello FZ. 2017 Abrupt species loss of the Amazonian dung beetle in pastures adjacent to species-rich forests. *J. Insect Conserv.* **21**, 487–494. (doi:10.1007/s10841-017-9988-9)

23. Byk A, Rutkiewicz A. 2017 Abundance and species structure of dung beetles in the regeneration cycle of Scots pine stands in Czluchów Forest (Pomeranian Lake District). *Sylwan* **161**, 781–792.

24. Watkins E, Kitching RL, Nakamura A, Stork NE. 2017 Beetle assemblages in rainforest gaps along a subtropical to tropical latitudinal gradient. *Biodivers. Conserv.* **26**, 1689–1703. (doi:10.1007/s10531-017-1326-8)

25. Cajaiba RL, Périco E, da Silva WB, Santos M. 2017 Can dung beetles (Scarabaeinae) indicate the status of Amazonia’s ecosystems? Insights integrating anthropogenic disturbance with seasonal patterns. *Anim. Biol.* **67**, 301–318. (doi:10.1163/15707563-00002538)

26. Rangel-Acosta JL, Martínez-Hernández NJ. 2017 Comparación de los ensamblajes de escarabajos copronecrófagos (Scarabaeidae: Scarabaeinae) entre fragmentos de bosque seco tropical y la matriz adyacente en el departamento del Atlántico-Colombia. *Rev. Mex. Biodivers.* **88**, 389–401. (doi:10.1016/j.rmb.2017.03.012)

27. Giménez Gómez VC, Lomáscolo SB, Zurita GA, Ocampo F. 2017 Daily Activity Patterns and Thermal Tolerance of Three Sympatric Dung Beetle Species (Scarabaeidae: Scarabaeinae: Eucraniini) from the Monte Desert, Argentina. *Neotrop. Entomol.* **47**, 821–827. (doi:10.1007/s13744-017-0567-2)

28. Batilani-Filho M, Hernandez MIM. 2017 Decline of Ecological Functions Performed by Dung Beetles in Areas of Atlantic Forest and Contribution of Rollers and Tunnellers in Organic Matter Removal. *Environ. Entomol.* **46**, 784–793. (doi:10.1093/ee/nvx091)

29. Labidi I, Nouira S, Errouissi F. 2017 Diversity and structure of dung beetle assemblages under two contrasted habitats in Tunisia: oases vs. humid pastures. *Austral Entomol.* **56**, 54–63. (doi:10.1111/aen.12210)

30. Audino LD, Murphy SJ, Zambaldi L, Louzada J, Comita LS. 2017 Drivers of community assembly in tropical forest restoration sites: role of local environment, landscape, and space. *Ecol. Appl.* **27**, 1731–1745. (doi:10.1002/eap.1562)

31. Beiroz W, Slade EM, Barlow J, Silveira JM, Louzada J, Sayer E. 2017 Dung beetle community dynamics in undisturbed tropical forests: implications for ecological evaluations of land-use change. *Insect Conserv. Divers.* **10**, 94–106. (doi:10.1111/icad.12206)

32. Farias PM de, Hernández MIM. 2017 Dung Beetles Associated with Agroecosystems of Southern Brazil: Relationship with Soil Properties. *Rev. Bras. Ciência do Solo* **41**. (doi:10.1590/18069657rbcs20160248)

33. Vieira L, Silva FAB, Louzada J. 2017 Dung beetles in a Caatinga Natural Reserve: a threatened Brazilian dry-forest with high biological value. *Iheringia. Série Zool.* **107**. (doi:10.1590/1678-4766e2017045)

34. Villada-Bedoya S, Cultid-Medina CA, Escobar F, Guevara R, Zurita G. 2017 Edge effects on dung beetle assemblages in an Andean mosaic of forest and coffee plantations. *Biotropica* **49**, 195–205. (doi:10.1111/btp.12373)

35. Salomão RP, Iannuzzi L. 2017 How Do Regeneration Stages of Caatinga Forests Influence the Structure of Dung Beetle (Coleoptera: Scarabaeidae) Assemblage? *Coleopt. Bull.* **71**, 578–588. (doi:10.1649/0010-065X-71.3.578)

36. França FM, Frazão FS, Korasaki V, Louzada J, Barlow J. 2017 Identifying thresholds of logging intensity on dung beetle communities to improve the sustainable management of Amazonian tropical forests. *Biol. Conserv.* **216**, 115–122. (doi:10.1016/j.biocon.2017.10.014)

37. Gómez-Cifuentes A, Munevar A, Gimenez VC, Gatti MG, Zurita GA. 2017 Influence of land use on the taxonomic and functional diversity of dung beetles (Coleoptera: Scarabaeinae) in the southern Atlantic forest of Argentina. *J. Insect Conserv.* **21**, 147–156. (doi:10.1007/s10841-017-9964-4)

38. Frank K, Hülsmann M, Assmann T, Schmitt T, Blüthgen N. 2017 Land use affects dung beetle communities and their ecosystem service in forests and grasslands. *Agric. Ecosyst. Environ.* **243**, 114–122. (doi:10.1016/j.agee.2017.04.010)

39. Edwards FA, Finan J, Graham LK, Larsen TH, Wilcove DS, Hsu WW, Chey VK, Hamer KC. 2017 The impact of logging roads on dung beetle assemblages in a tropical rainforest reserve. *Biol. Conserv.* **205**, 85–92. (doi:10.1016/j.biocon.2016.11.011)

40. Costa C, Oliveira VHF, Maciel R, Beiroz W, Korasaki V, Louzada J. 2017 Variegated tropical landscapes conserve diverse dung beetle communities. *PeerJ.* **5**, e3125. (doi:10.7717/peerj.3125)

41. van Schalkwyk J, Pryke JS, Samways MJ. 2017 Wide corridors with much environmental heterogeneity best conserve high dung beetle and ant diversity. *Biodivers. Conserv.* **26**, 1243–1256. (doi:10.1007/s10531-017-1299-7)

42. Gray CL, Simmons BI, Fayle TM, Mann DJ, Slade EM. 2016 Are riparian forest reserves sources of invertebrate biodiversity spillover and associated ecosystem functions in oil palm landscapes? *Biol. Conserv.* **194**, 176–183. (doi:10.1016/j.biocon.2015.12.017)

43. Sabu TK, Nithya S. 2016 Comparison of the Arboreal Dung Beetles (Coleoptera: Scarabaeidae: Scarabaeinae) of the Wet and Dry Forests of the Western Ghats, India. *Coleopt. Bull.* **70**, 144–148. (doi:10.1649/072.070.0121)

44. Abdel-Dayem MS, Kondratieff BC, Fadl HH, Al Dhafer HM. 2016 Dung Beetle (Coleoptera: Scarabaeidae) Abundance and Diversity at Nature Preserve Within Hyper-Arid Ecosystem of Arabian Peninsula. *Ann. Entomol. Soc. Am.* **109**, 216–223. (doi:10.1093/aesa/sav154)

45. Silva RJ, Storck-Tonon D, Vaz-de-Mello FZ. 2016 Dung beetle (Coleoptera: Scarabaeinae) persistence in Amazonian forest fragments and adjacent pastures: biogeographic implications for alpha and beta diversity. *J. Insect Conserv.* **20**, 549–564. (doi:10.1007/s10841-016-9885-7)

46. Tocco C, Villet M. 2016 Dung beetle (Coleoptera: Scarabaeoidea) assemblages in the western Italian Alps: benchmark data for land use monitoring. *Biodivers. Data J.* **4**, e10059. (doi:10.3897/BDJ.4.e10059)

47. Nunes CA, Braga RF, Figueira JEC, Neves F de S, Fernandes GW. 2016 Dung Beetles along a Tropical Altitudinal Gradient: Environmental Filtering on Taxonomic and Functional Diversity. *PLoS One* **11**, e0157442. (doi:10.1371/journal.pone.0157442)

48. Martello F, Andriolli F, de Souza TB, Dodonov P, Ribeiro MC. 2016 Edge and land use effects on dung beetles (Coleoptera: Scarabaeidae: Scarabaeinae) in Brazilian cerrado vegetation. *J. Insect Conserv.* **20**, 957–970. (doi:10.1007/s10841-016-9928-0)

49. Sánchez-de-Jesús HA, Arroyo-Rodríguez V, Andresen E, Escobar F. 2016 Forest loss and matrix composition are the major drivers shaping dung beetle assemblages in a fragmented rainforest. *Landsc. Ecol.* **31**, 843–854. (doi:10.1007/s10980-015-0293-2)

50. Bitencourt BS, da Silva PG. 2016 Forest regeneration affects dung beetle assemblages (Coleoptera: Scarabaeinae) in the southern Brazilian Atlantic Forest. *J. Insect Conserv.* **20**, 855–866. (doi:10.1007/s10841-016-9917-3)

51. Bourg A, Escobar F, MacGregor-Fors I, Moreno CE. 2016 Got Dung? Resource Selection by Dung Beetles in Neotropical Forest Fragments and Cattle Pastures. *Neotrop. Entomol.* **45**, 490–498. (doi:10.1007/s13744-016-0397-7)

52. Hewavithana DK, Wijesinghe MR, Dangalle CD, Dharmarathne HASG. 2016 Habitat and dung preferences of scarab beetles of the subfamily Scarabaeinae: a case study in a tropical monsoon forest in Sri Lanka. *Int. J. Trop. Insect Sci.* **36**, 97–105. (doi:10.1017/S1742758416000023)

53. Novais SMA, Evangelista LA, Reis-Júnior R, Neves FS. 2016 How Does Dung Beetle (Coleoptera: Scarabaeidae) Diversity Vary Along a Rainy Season in a Tropical Dry Forest? *J. Insect Sci.* **16**, 81. (doi:10.1093/jisesa/iew069)

54. Ferrer-Paris JR, Lozano C, Cardozo-Urdaneta A, Thomas Cabianca A. 2016 Indicative response of Oxysternon festivum Linné (Coleoptera: Scarabaidae) to vegetation condition in the basin of the Orinoco river, Venezuela. *J. Insect Conserv.* **20**, 527–538. (doi:10.1007/s10841-016-9886-6)

55. Montoya-Molina S, Giraldo-Echeverri C, Montoya-Lerma J, Chará J, Escobar F, Calle Z. 2016 Land sharing vs. land sparing in the dry Caribbean lowlands: A dung beetles’ perspective. *Appl. Soil Ecol.* **98**, 204–212. (doi:10.1016/j.apsoil.2015.10.017)

56. Moctezuma V, Halffter G, Escobar F. 2016 Response of copronecrophagous beetle communities to habitat disturbance in two mountains of the Mexican Transition Zone: influence of historical and ecological factors. *J. Insect Conserv.* **20**, 945–956. (doi:10.1007/s10841-016-9923-5)

57. Filgueiras BKC, Tabarelli M, Leal IR, Vaz-de-Mello FZ, Peres CA, Iannuzzi L. 2016 Spatial replacement of dung beetles in edge-affected habitats: biotic homogenization or divergence in fragmented tropical forest landscapes? *Divers. Distrib.* **22**, 400–409. (doi:10.1111/ddi.12410)

58. da Silva PG, Hernández MIM. 2016 Spatial variation of dung beetle assemblages associated with forest structure in remnants of southern Brazilian Atlantic Forest. *Rev. Bras. Entomol.* **60**, 73–81. (doi:10.1016/j.rbe.2015.11.001)

59. Kenyon TM, Mayfield MM, Monteith GB, Menéndez R. 2016 The effects of land use change on native dung beetle diversity and function in Australia’s Wet Tropics. *Austral Ecol.* **41**, 797–808. (doi:10.1111/aec.12366)

60. Pryke JS, Roets F, Samways MJ. 2016 Wild Herbivore Grazing Enhances Insect Diversity over Livestock Grazing in an African Grassland System. *PLoS One* **11**, e0164198. (doi:10.1371/journal.pone.0164198)

61. Bogoni JA *et al.* 2016 Contributions of the mammal community, habitat structure, and spatial distance to dung beetle community structure. *Biodivers. Conserv.* **25**, 1661–1675. (doi:10.1007/s10531-016-1147-1)

62. Griffiths HM, Louzada J, Bardgett RD, Beiroz W, França F, Tregidgo D, Barlow J. 2015 Biodiversity and environmental context predict dung beetle-mediated seed dispersal in a tropical forest field experiment. *Ecology* **96**, 1607–1619. (doi:10.1890/14-1211.1)

63. Campos RRC, Hernández MIMM. 2015 Changes in the dynamics of functional groups in communities of dung beetles in Atlantic forest fragments adjacent to transgenic maize crops. *Ecol. Indic.* **49**, 216–227. (doi:10.1016/j.ecolind.2014.09.043)

64. Petre C-A, Zinque M-H, Tagg N, Beudels-Jamar R-C, Haurez B, Josso J-F, Moretto P, Doucet J-L. 2015 Differences in dung beetle activity at western gorilla defecation sites in south-east Cameroon: implications for establishment of Uapaca spp. seedlings. *J. Trop. Ecol.* **31**, 165–174. (doi:10.1017/S0266467414000753)

65. Salomão RP, Iannuzzi L. 2015 Dung beetle (Coleoptera, Scarabaeidae) assemblage of a highly fragmented landscape of Atlantic forest: from small to the largest fragments of northeastern Brazilian region. *Rev. Bras. Entomol.* **59**, 126–131. (doi:10.1016/j.rbe.2015.03.008)

66. Filgueiras BKCC, Tabarelli M, Leal IR, Vaz-de-Mello FZ, Iannuzzi L. 2015 Dung beetle persistence in human-modified landscapes: Combining indicator species with anthropogenic land use and fragmentation-related effects. *Ecol. Indic.* **55**, 65–73. (doi:10.1016/j.ecolind.2015.02.032)

67. Ueda A, Dwibadra D, Noerdjito WA, Sugiarto, Kon M, Ochi T, Takahashi M, Fukuyama K. 2015 Effect of habitat transformation from grassland to Acacia mangium plantation on dung beetle assemblage in East Kalimantan, Indonesia. *J. Insect Conserv.* **19**, 765–780. (doi:10.1007/s10841-015-9798-x)

68. Tissiani ASO, Sousa WO, Santos GB, Ide S, Battirola L, Marques MI. 2015 Environmental influence on coprophagous Scarabaeidae (Insecta, Coleoptera) assemblages in the Pantanal of Mato Grosso. *Brazilian J. Biol.* **75**, 136–142. (doi:10.1590/1519-6984.07514)

69. Ibarra-Polesel MG, Damborsky MP, Porcel E. 2015 Escarabajos copronecrófagos (Scarabaeidae: Scarabaeinae) de la Reserva Natural Educativa Colonia Benítez, Chaco, Argentina. *Rev. Mex. Biodivers.* **86**, 744–753. (doi:10.1016/j.rmb.2015.05.011)

70. de Lima JDN, Silva V da C, Bianchi V, da Silva PG, Mare RA Di. 2015 Estrutura e organização de assembleias de Scarabaeinae (Coleoptera, Scarabaeidae) em diferentes fitofisionomias no sul do Brasil. *Iheringia. Série Zool.* **105**, 393–402. (doi:10.1590/1678-476620151054393402)

71. Silva RJ, Ribeiro HV., Souza MF, Vaz-De-Mello FZ. 2015 Influência da granulometria do solo na estrutura de guildas funcionais de besouros rola-bostas (Coleoptera: Scarabaeidae: Scarabaeinae) em florestas semideciduais no estado do mato Grosso, Brasil. *Biosci. J.* **31**, 601–612. (doi:10.14393/BJ-v31n1a2015-23525)

72. Chamberlain D, Tocco C, Longoni A, Mammola S, Palestrini C, Rolando A. 2015 Nesting strategies affect altitudinal distribution and habitat use in Alpine dung beetle communities. *Ecol. Entomol.* **40**, 372–380. (doi:10.1111/een.12195)

73. Buse J, Šlachta M, Sladecek FXJ, Pung M, Wagner T, Entling MH. 2015 Relative importance of pasture size and grazing continuity for the long-term conservation of European dung beetles. *Biol. Conserv.* **187**, 112–119. (doi:10.1016/j.biocon.2015.04.011)

74. de Farias PM, Arellano L, Hernández MIM, Ortiz SL. 2015 Response of the copro-necrophagous beetle (Coleoptera: Scarabaeinae) assemblage to a range of soil characteristics and livestock management in a tropical landscape. *J. Insect Conserv.* **19**, 947–960. (doi:10.1007/s10841-015-9812-3)

75. da Silva PG, Hernández MIM, da Silva PG, Hernández MIM. 2015 Scale-Dependence of Processes Structuring Dung Beetle Metacommunities Using Functional Diversity and Community Deconstruction Approaches. *PLoS One* **10**, e0123030. (doi:10.1371/journal.pone.0123030)

76. Damborsky MP, Alvarez Bohle MC, Ibarra Polesel MG, Porcel EA, Fontana JL. 2015 Spatial and Temporal Variation of Dung Beetle Assemblages in a Fragmented Landscape at Eastern Humid Chaco. *Neotrop. Entomol.* **44**, 30–39. (doi:10.1007/s13744-014-0257-2)

77. Campos RC, Hernández MIM. 2015 The Importance of Maize Management on Dung Beetle Communities in Atlantic Forest Fragments. *PLoS One* **10**, e0145000. (doi:10.1371/journal.pone.0145000)

78. Byk A, Węgrzynowicz P. 2015 The Structure and Seasonal Dynamics of Coprophagous Scarabaeoidea (Coleoptera) Communities in Later Developmental Stages of Pine Stands in NW Poland. *J. Entomol. Res. Soc.* **17**, 39–57.

79. da Costa CMQ, Barretto JW, de Moura RDC. 2014 Changes in the dung beetle community in response to restinga forest degradation. *J. Insect Conserv.* **18**, 895–902. (doi:10.1007/s10841-014-9697-6)

80. Menéndez R, González-Megías A, Jay-Robert P, Marquéz-Ferrando R. 2014 Climate change and elevational range shifts: evidence from dung beetles in two European mountain ranges. *Glob. Ecol. Biogeogr.* **23**, 646–657. (doi:10.1111/geb.12142)

81. Puker A, Correa CMA, Korasaki V. 2014 Deltochilini and Phanaeini dung beetles (Coleoptera: Scarabaeidae: Scarabaeinae) in introduced and native ecosystems of Brazil. *J. Nat. Hist.* **48**, 2105–2116. (doi:10.1080/00222933.2014.908969)

82. Gray CL, Slade EM, Mann DJ, Lewis OT. 2014 Do riparian reserves support dung beetle biodiversity and ecosystem services in oil palm-dominated tropical landscapes? *Ecol. Evol.* **4**, 1049–1060. (doi:10.1002/ece3.1003)

83. Viegas G, Stenert C, Schulz UH, Maltchik L. 2014 Dung beetle communities as biological indicators of riparian forest widths in southern Brazil. *Ecol. Indic.* **36**, 703–710. (doi:10.1016/j.ecolind.2013.09.036)

84. Bicknell JE, Phelps SP, Davies RG, Mann DJ, Struebig MJ, Davies ZG. 2014 Dung beetles as indicators for rapid impact assessments: Evaluating best practice forestry in the neotropics. *Ecol. Indic.* **43**, 154–161. (doi:10.1016/j.ecolind.2014.02.030)

85. Audino LD, Louzada J, Comita L. 2014 Dung beetles as indicators of tropical forest restoration success: Is it possible to recover species and functional diversity? *Biol. Conserv.* **169**, 248–257. (doi:10.1016/j.biocon.2013.11.023)

86. Gilroy JJ, Edwards FA, Medina Uribe CA, Haugaasen T, Edwards DP. 2014 EDITOR’S CHOICE: Surrounding habitats mediate the trade-off between land-sharing and land-sparing agriculture in the tropics. *J. Appl. Ecol.* **51**, 1337–1346. (doi:10.1111/1365-2664.12284)

87. Doll HHM, Butod E, Harrison RD, Fletcher C, Kassim AR, Ibrahim S, Potts MD. 2014 Environmental and geographic factors driving dung beetle (Coleoptera: Scarabaeidae: Scarabaeinae) diversity in the dipterocarp forests of Peninsular Malaysia. *Raffles Bull. Zool.* **62**, 549–560.

88. Hosaka T, Niino M, Kon M, Ochi T, Yamada T, Fletcher CD, Okuda T. 2014 Impacts of Small-scale Clearings due to Selective logging on Dung Beetle Communities. *Biotropica* **46**, 720–731. (doi:10.1111/btp.12158)

89. Imura O, Morimoto N, Shi K, Sasaki H. 2014 Landscape diversity of pasture dung beetle communities in the central region of mainland Japan and implications for conservation management. *Biodivers. Conserv.* **23**, 597–616. (doi:10.1007/s10531-014-0619-4)

90. da Silva PG, Hernández MIM. 2014 Local and Regional Effects on Community Structure of Dung Beetles in a Mainland-Island Scenario. *PLoS One* **9**, e111883. (doi:10.1371/journal.pone.0111883)

91. Barnes AD, Emberson RM, Chapman HM, Krell F-T, Didham RK. 2014 Matrix habitat restoration alters dung beetle species responses across tropical forest edges. *Biol. Conserv.* **170**, 28–37. (doi:10.1016/j.biocon.2013.12.006)

92. Hernández MIM, Barreto PSCS, Costa VH, Creão-Duarte AJ, Favila ME. 2014 Response of a dung beetle assemblage along a reforestation gradient in Restinga forest. *J. Insect Conserv.* **18**, 539–546. (doi:10.1007/s10841-014-9645-5)

93. Silva RJ, Coletti F, Costa DA, Vaz-De-Mello FZ. 2014 Rola-bostas (Coleoptera: Scarabaeidae: Scarabaeinae) de florestas e pastagens no sudoeste da Amazônia brasileira: Levantamento de espécies e guildas alimentares. *Acta Amaz.* **44**, 345–352. (doi:10.1590/1809-4392201304472)

94. Davis ALV, Swemmer AM, Scholtz CH, Deschodt CM, Tshikae BP. 2014 Roles of environmental variables and land usage as drivers of dung beetle assemblage structure in mopane woodland. *Austral Ecol.* **39**, 313–327. (doi:10.1111/aec.12081)

95. Wassmer T. 2014 Seasonal Occurrence (Phenology) of Coprophilous Beetles (Coleoptera: Scarabaeidae and Hydrophilidae) from Cattle and Sheep Farms in Southeastern Michigan, USA. *Coleopt. Bull.* **68**, 603–618. (doi:10.1649/072.068.0327)

96. Medina AM, Lopes PP. 2014 Seasonality in the dung beetle community in a Brazilian tropical dry forest: Do small changes make a difference? *J. Insect Sci.* **14**, 123. (doi:10.1093/jis/14.1.123)

97. Daniel GM, Nunes LGOA, Vaz-de-Mello FZ. 2014 Species composition and functional guilds of dung beetles (Insecta: Coleoptera: Scarabaeidae: Scarabaeinae) in different vegetational types in the Brazilian Shield–Chacoan Depression Border. *Ann. la Société Entomol. Fr.* **50**, 183–190. (doi:10.1080/00379271.2014.938936)

98. Barragán F, Moreno CE, Escobar F, Bueno-Villegas J, Halffter G. 2014 The impact of grazing on dung beetle diversity depends on both biogeographical and ecological context. *J. Biogeogr.* **41**, 1991–2002. (doi:10.1111/jbi.12351)

99. Plexida SG. 2014 The impact of human land use on the composition and richness of ground and dung beetle assemblages. *Appl. Ecol. Environ. Res.* **12**, 661–679. (doi:10.15666/aeer/1203_661679)

100. Sheldon KS, Tewksbury JJ. 2014 The impact of seasonality in temperature on thermal tolerance and elevational range size. *Ecology* **95**, 2134–2143. (doi:10.1890/13-1703.1)

101. Barnes AD, Emberson RM, Krell F, Didham RK. 2014 The Role of Species Traits in Mediating Functional Recovery during Matrix Restoration. *PLoS One* **9**, e115385. (doi:10.1371/journal.pone.0115385)

102. de Andrade RB, Barlow J, Louzada J, Vaz-de-Mello FZ, Silveira JM, Cochrane MA. 2014 Tropical forest fires and biodiversity: dung beetle community and biomass responses in a northern Brazilian Amazon forest. *J. Insect Conserv.* **18**, 1097–1104. (doi:10.1007/s10841-014-9719-4)

103. Arellano L, León-Cortés JL, Halffter G, Montero J. 2013 Acacia woodlots, cattle and dung beetles (Coleoptera: Scarabaeinae) in a Mexican silvopastoral landscape. *Rev. Mex. Biodivers.* **84**, 650–660. (doi:10.7550/rmb.32911)

104. Korasaki V, Braga RF, Zanetti R, Moreira FMS, Vaz-de-Mello FZ, Louzada J. 2013 Conservation value of alternative land-use systems for dung beetles in Amazon: valuing traditional farming practices. *Biodivers. Conserv.* **22**, 1485–1499. (doi:10.1007/s10531-013-0487-3)

105. Tocco C, Negro M, Rolando A, Palestrini C. 2013 Does natural reforestation represent a potential threat to dung beetle diversity in the Alps? *J. Insect Conserv.* **17**, 207–217. (doi:10.1007/s10841-012-9498-8)

106. Tshikae BP, Davis ALV V., Scholtz CH. 2013 Dung beetle assemblage structure across the aridity and trophic resource gradient of the Botswana Kalahari: Patterns and drivers at regional and local scales. *J. Insect Conserv.* **17**, 623–636. (doi:10.1007/s10841-013-9547-y)

107. Campos RC, Hernández MIM. 2013 Dung beetle assemblages (Coleoptera, Scarabaeinae) in Atlantic forest fragments in southern Brazil. *Rev. Bras. Entomol.* **57**, 47–54. (doi:10.1590/S0085-56262013000100008)

108. Braga RF, Korasaki V, Andresen E, Louzada J. 2013 Dung Beetle Community and Functions along a Habitat-Disturbance Gradient in the Amazon: A Rapid Assessment of Ecological Functions Associated to Biodiversity. *PLoS One* **8**, e57786. (doi:10.1371/journal.pone.0057786)

109. Rodrigues M, Uchôa M, Ide S. 2013 Dung beetles (Coleoptera: Scarabaeoidea) in three landscapes in Mato Grosso do Sul, Brazil. *Brazilian J. Biol.* **73**, 211–220. (doi:10.1590/S1519-69842013000100023)

110. Mason NWH, de Bello F. 2013 Functional diversity: a tool for answering challenging ecological questions. *J. Veg. Sci.* **24**, 777–780. (doi:10.1111/jvs.12097)

111. Pryke JS, Roets F, Samways MJ. 2013 Importance of habitat heterogeneity in remnant patches for conserving dung beetles. *Biodivers. Conserv.* **22**, 2857–2873. (doi:10.1007/s10531-013-0559-4)

112. Tocco C, Probo M, Lonati M, Lombardi G, Negro M, Nervo B, Rolando A, Palestrini C. 2013 Pastoral Practices to Reverse Shrub Encroachment of Sub-Alpine Grasslands: Dung Beetles (Coleoptera, Scarabaeoidea) Respond More Quickly Than Vegetation. *PLoS One* **8**, e83344. (doi:10.1371/journal.pone.0083344)

113. Peyras M, Vespa NI, Bellocq MI, Zurita GA. 2013 Quantifying edge effects: the role of habitat contrast and species specialization. *J. Insect Conserv.* **17**, 807–820. (doi:10.1007/s10841-013-9563-y)

114. Nichols E *et al.* 2013 Trait-dependent response of dung beetle populations to tropical forest conversion at local and regional scales. *Ecology* **94**, 180–189. (doi:10.1890/12-0251.1)

115. Korasaki V, Lopes J, Gardner Brown G, Louzada J. 2013 Using dung beetles to evaluate the effects of urbanization on Atlantic Forest biodiversity. *Insect Sci.* **20**, 393–406. (doi:10.1111/j.1744-7917.2012.01509.x)

116. Feer F. 2013 Variations in dung beetles assemblages (Coleoptera: Scarabaeidae) within two rain forest habitats in French Guiana. *Rev. Biol. Trop.* **61**, 753–68.

117. Costa FC, Pessoa KKT, Liberal CN, Filgueiras BKC, Salomão RP, Iannuzzi L. 2013 What is the importance of open habitat in a predominantly closed forest area to the dung beetle (Coleoptera, Scarabaeinae) assemblage? *Rev. Bras. Entomol.* **57**, 329–334. (doi:10.1590/S0085-56262013000300012)

118. Abot AR, Puker A, Taira TL, Rodrigues SR, Korasaki V, de Oliveira HN. 2012 Abundance and diversity of coprophagous beetles (Coleoptera: Scarabaeidae) caught with a light trap in a pasture area of the Brazilian Cerrado. *Stud. Neotrop. Fauna Environ.* **47**, 53–60. (doi:10.1080/01650521.2012.662846)

119. Numa C, Verdú JR, Rueda C, Galante E. 2012 Comparing Dung Beetle Species Assemblages Between Protected Areas and Adjacent Pasturelands in a Mediterranean Savanna Landscape. *Rangel. Ecol. Manag.* **65**, 137–143. (doi:10.2111/REM-D-10-00050.1)

120. Agoglitta R, Moreno CE, Zunino M, Bonsignori G, Dellacasa M. 2012 Cumulative annual dung beetle diversity in Mediterranean seasonal environments. *Ecol. Res.* **27**, 387–395. (doi:10.1007/s11284-011-0910-8)

121. Davis ALV, Scholtz CH, Swemmer AM. 2012 Effects of land usage on dung beetle assemblage structure: Kruger National Park versus adjacent farmland in South Africa. *J. Insect Conserv.* **16**, 399–411. (doi:10.1007/s10841-011-9426-3)

122. Rös M, Escobar F, Halffter G. 2012 How dung beetles respond to a human-modified variegated landscape in Mexican cloud forest: a study of biodiversity integrating ecological and biogeographical perspectives. *Divers. Distrib.* **18**, 377–389. (doi:10.1111/j.1472-4642.2011.00834.x)

123. Edwards DP, Backhouse AR, Wheeler C, Khen CV, Hamer KC. 2012 Impacts of logging and rehabilitation on invertebrate communities in tropical rainforests of northern Borneo. *J. Insect Conserv.* **16**, 591–599. (doi:10.1007/s10841-011-9444-1)

124. Numa C, Lobo JM, Verdú JR. 2012 Scaling local abundance determinants in Mediterranean dung beetles. *Insect Conserv. Divers.* **5**, 106–117. (doi:10.1111/j.1752-4598.2011.00137.x)

125. Labidi I, Errouissi F, Nouira S. 2012 Spatial and Temporal Variation in Species Composition, Diversity, and Structure of Mediterranean Dung Beetle Assemblages (Coleoptera: Scarabaeidae) Across a Bioclimatic Gradient. *Environ. Entomol.* **41**, 785–801. (doi:10.1603/EN11278)

126. Larsen TH. 2012 Upslope Range Shifts of Andean Dung Beetles in Response to Deforestation: Compounding and Confounding Effects of Microclimatic Change. *Biotropica* **44**, 82–89. (doi:10.1111/j.1744-7429.2011.00768.x)

127. Lopes J, Korasaki V, Catelli LL, Marçal VVM, Nunes MPBP. 2011 A comparison of dung beetle assemblage structure (Coleoptera: Scarabaeidae: Scarabaeinae) between an Atlantic forest fragment and adjacent abandoned pasture in Paraná, Brazil. *Zool.* **28**, 72–79. (doi:10.1590/S1984-46702011000100011)

128. Slade EM, Mann DJ, Lewis OT. 2011 Biodiversity and ecosystem function of tropical forest dung beetles under contrasting logging regimes. *Biol. Conserv.* **144**, 166–174. (doi:10.1016/j.biocon.2010.08.011)

129. da Silva P. 2011 Dung beetles (Coleoptera: Scarabaeidae: Scarabaeinae) of two non-native habitats in Bagé, Rio Grande do Sul, Brazil. *Zool. Stud.* **50**, 546–559.

130. Kunz BK, Krell F-TT. 2011 Habitat differences in dung beetle assemblages in an African savanna-forest ecotone: implications for secondary seed dispersal. *Integr. Zool.* **6**, 81–96. (doi:10.1111/j.1749-4877.2011.00240.x)

131. Filgueiras BKC, Iannuzzi L, Leal IR. 2011 Habitat fragmentation alters the structure of dung beetle communities in the Atlantic Forest. *Biol. Conserv.* **144**, 362–369. (doi:10.1016/j.biocon.2010.09.013)

132. Liberal CN, Farias ÂMI de, Meiado MV, Filgueiras BKC, Iannuzzi L. 2011 How Habitat Change and Rainfall Affect Dung Beetle Diversity in Caatinga, a Brazilian Semi-Arid Ecosystem. *J. Insect Sci.* **11**, 1–11. (doi:10.1673/031.011.11401)

133. Barragán F, Moreno C, Escobar F. 2011 Negative impacts of human land use on dung beetle functional diversity. *PLoS One* **6**, e17976. (doi: 10.1371/journal.pone.0017976)

134. Andrade RB de, Barlow J, Louzada J, Vaz-de-Mello FZ, Souza M, Silveira JM, Cochrane MA. 2011 Quantifying Responses of Dung Beetles to Fire Disturbance in Tropical Forests: The Importance of Trapping Method and Seasonality. *PLoS One* **6**, e26208. (doi:10.1371/journal.pone.0026208)

135. Audino LD, Silva PG da, Nogueira JM, Moraes LP de, Vaz-de-Mello FZ. 2011 Scarabaeinae (Coleoptera, Scarabaeidae) de um bosque de eucalipto introduzido em uma região originalmente campestre. *Iheringia. Série Zool.* **101**, 121–126. (doi:10.1590/S0073-47212011000100017)

136. Almeida S, Louzada J, Sperber C, Barlow J. 2011 Subtle Land-Use Change and Tropical Biodiversity: Dung Beetle Communities in Cerrado Grasslands and Exotic Pastures. *Biotropica* **43**, 704–710. (doi:10.1111/j.1744-7429.2011.00751.x)

137. Negro M, Palestrini C, Giraudo MT, Rolando A. 2011 The effect of local environmental heterogeneity on species diversity of alpine dung beetles (Coleoptera: Scarabaeidae). *Eur. J. Entomol.* **108**, 91–98. (doi:10.14411/eje.2011.012)

138. Negro M, Rolando A, Palestrini C. 2011 The Impact of Overgrazing on Dung Beetle Diversity in the Italian Maritime Alps. *Environ. Entomol.* **40**, 1081–1092. (doi:10.1603/EN11105)

139. Verdú JR, Numa C, Hernández-Cuba O. 2011 The influence of landscape structure on ants and dung beetles diversity in a Mediterranean savanna - Forest ecosystem. *Ecol. Indic.* **11**, 831–839. (doi:10.1016/j.ecolind.2010.10.011)

140. Gollan JR, Reid C a M, Barnes PB, Wilkie L. 2011 The ratio of exotic-to-native dung beetles can indicate habitat quality in riparian restoration. *Insect Conserv. Divers.* **4**, 123–131. (doi:10.1111/j.1752-4598.2010.00115.x)

141. Louzada J, Lima AP, Matavelli R, Zambaldi L, Barlow J. 2010 Community structure of dung beetles in Amazonian savannas: role of fire disturbance, vegetation and landscape structure. *Landsc. Ecol.* **25**, 631–641. (doi:10.1007/s10980-010-9448-3)

142. da Silva R, Diniz S, Vaz-de-Mello FZ. 2010 Habitat heterogeneity, richness and structure of assemblages of dung beetles (Scarabaeidae: Scarabaeinae) in areas of cerrado in the Chapada dos Parecis, Mato Grosso state, Brazil. *Neotrop. Entomol.* **39**, 934–941. (doi:10.1590/S1519-566X2010000600014)

143. Jacobs CT, Scholtz CH, Escobar F, Davis AL V. 2010 How might intensification of farming influence dung beetle diversity (Coleoptera: Scarabaeidae) in Maputo Special Reserve (Mozambique)? *J. Insect Conserv.* **14**, 389–399. (doi:10.1007/s10841-010-9270-x)

144. Silva FAB, Costa CMQ, Moura RC, Farias AI. 2010 Study of the Dung Beetle (Coleoptera: Scarabaeidae) Community at Two Sites: Atlantic Forest and Clear-Cut, Pernambuco, Brazil. *Environ. Entomol.* **39**, 359–367. (doi:10.1603/EN09180)

145. de Siqueira Neves F, Hugo Fonseca Oliveira V, Marcos do Espírito-Santo M, Zagury Vaz-de-Mello F, Louzada J, Sanchez-Azofeifa A, Wilson Fernandes G. 2010 Successional and Seasonal Changes in a Community of Dung Beetles (Coleoptera: Scarabaeinae) in a Brazilian Tropical Dry Forest. *Nat. Conserv.* **08**, 160–164. (doi:10.4322/natcon.00802009)

146. Tregidgo DJ, Qie L, Barlow J, Sodhi NS, Lim SL-H. 2010 Vertical Stratification Responses of an Arboreal Dung Beetle Species to Tropical Forest Fragmentation in Malaysia. *Biotropica* **42**, 521–525. (doi:10.1111/j.1744-7429.2010.00649.x)

147. Barlow J, Louzada J, Parry L, Hernández MIM, Hawes J, Peres CA, Vaz-de-Mello FZ, Gardner TA. 2010 Improving the design and management of forest strips in human-dominated tropical landscapes: A field test on Amazonian dung beetles. *J. Appl. Ecol.* **47**, 779–788. (doi:10.1111/j.1365-2664.2010.01825.x)

148. Nyeko P. 2009 Dung Beetle Assemblages and Seasonality in Primary Forest and Forest Fragments on Agricultural Landscapes in Budongo, Uganda. *Biotropica* **41**, 476–484. (doi:10.1111/j.1744-7429.2009.00499.x)

149. Numa C, Verdú JR, Sánchez A, Galante E. 2009 Effect of landscape structure on the spatial distribution of Mediterranean dung beetle diversity. *Divers. Distrib.* **15**, 489–501. (doi:10.1111/j.1472-4642.2009.00559.x)

150. Masís A, Marquis RJ. 2009 Effects of even-aged and uneven-aged timber management on dung beetle community attributes in a Missouri Ozark forest. *For. Ecol. Manage.* **257**, 536–545. (doi:10.1016/j.foreco.2008.09.036)

151. Horgan F. 2009 Invasion and retreat: shifting assemblages of dung beetles amidst changing agricultural landscapes in central Peru. *Biodivers. Conserv.* **18**, 3519–3541. (doi:10.1007/s10531-009-9658-7)

152. Hayes L, Mann DJ, Monastyrskii AL, Lewis OT. 2009 Rapid assessments of tropical dung beetle and butterfly assemblages: contrasting trends along a forest disturbance gradient. *Insect Conserv. Divers.* **2**, 194–203. (doi:10.1111/j.1752-4598.2009.00058.x)

153. Carpio C, Donoso DA, Ramón G, Dangles O. 2009 Short term response of dung beetle communities to disturbance by road construction in the Ecuadorian Amazon. *Ann. la Société Entomol. Fr.* **45**, 455–469. (doi:10.1080/00379271.2009.10697629)

154. Roslin T, Avomaa T, Leonard M, Luoto M, Ovaskainen O. 2009 Some like it hot: microclimatic variation affects the abundance and movements of a critically endangered dung beetle. *Insect Conserv. Divers.* **2**, 232–241. (doi:10.1111/j.1752-4598.2009.00054.x)

155. Macagno ALM, Palestrini C. 2009 The maintenance of extensively exploited pastures within the Alpine mountain belt: implications for dung beetle conservation (Coleoptera: Scarabaeoidea). *Biodivers. Conserv.* **18**, 3309–3323. (doi:10.1007/s10531-009-9643-1)

156. Navarrete D, Halffter G. 2008 Dung beetle (Coleoptera: Scarabaeidae: Scarabaeinae) diversity in continuous forest, forest fragments and cattle pastures in a landscape of Chiapas, Mexico: the effects of anthropogenic changes. *Biodivers. Conserv.* **17**, 2869–2898. (doi:10.1007/s10531-008-9402-8)

157. Horgan FG. 2008 Dung beetle assemblages in forests and pastures of El Salvador: a functional comparison. *Biodivers. Conserv.* **17**, 2961–2978. (doi:10.1007/s10531-008-9408-2)

158. Andresen E. 2008 Dung beetle assemblages in primary forest and disturbed habitats in a tropical dry forest landscape in western Mexico. *J. Insect Conserv.* **12**, 639–650. (doi:10.1007/s10841-007-9100-y)

159. Vulinec K, Pimentel A, Carvalho EAR, Mellow DJ. 2008 Dung Beetles and Long-term Habitat Fragmentation in Alter do Chão, Amazônia, Brazil. *Trop. Conserv. Sci.* **1**, 111–121. (doi:10.1177/194008290800100204)

160. Vieira L, Louzada JNC, Spector S. 2008 Effects of Degradation and Replacement of Southern Brazilian Coastal Sandy Vegetation on the Dung Beetles (Coleoptera: Scarabaeidae). *Biotropica* **40**, 719–727. (doi:10.1111/j.1744-7429.2008.00432.x)

161. Davis ALV, Scholtz CH, Deschodt C. 2008 Multi-scale determinants of dung beetle assemblage structure across abiotic gradients of the Kalahari-Nama Karoo ecotone, South Africa. *J. Biogeogr.* **35**, 1465–1480. (doi:10.1111/j.1365-2699.2007.01872.x)

162. Jay-Robert P, Niogret J, Errouissi F, Labarussias M, Paoletti É, Luis MV, Lumaret J-P. 2008 Relative efficiency of extensive grazing vs. wild ungulates management for dung beetle conservation in a heterogeneous landscape from Southern Europe (Scarabaeinae, Aphodiinae, Geotrupinae). *Biol. Conserv.* **141**, 2879–2887. (doi:10.1016/j.biocon.2008.09.001)

163. Arellano L, León-Cortés JL, Halffter G. 2008 Response of dung beetle assemblages to landscape structure in remnant natural and modified habitats in southern Mexico. *Insect Conserv. Divers.* **1**, 253–262. (doi:10.1111/j.1752-4598.2008.00033.x)

164. Jay-Robert P, Lumaret J-P, Lebreton J-D. 2008 Spatial and Temporal Variation of Mountain Dung Beetle Assemblages and Their Relationships with Environmental Factors (Aphodiinae: Geotrupinae: Scarabaeinae). *Ann. Entomol. Soc. Am.* **101**, 58–69. (doi:10.1603/0013-8746(2008)101[58:SATVOM]2.0.CO;2)

165. Jay-Robert P, Errouissi F, Lumaret JP. 2008 Temporal coexistence of dung-dweller and soil-digger dung beetles (Coleoptera, Scarabaeoidea) in contrasting Mediterranean habitats. *Bull. Entomol. Res.* **98**. (doi:10.1017/S0007485307005615)

166. Larsen TH, Lopera A, Forsyth A. 2008 Understanding trait-dependent community disassembly: Dung beetles, density functions, and forest fragmentation. *Conserv. Biol.* **22**, 1288–1298. (doi:10.1111/j.1523-1739.2008.00969.x)

167. Aguilar-Amuchastegui N, Henebry GM. 2007 Assessing sustainability indicators for tropical forests: Spatio-temporal heterogeneity, logging intensity, and dung beetle communities. *For. Ecol. Manage.* **253**, 56–67. (doi:10.1016/j.foreco.2007.07.004)

168. Novelo ER, Delfín-González H, Morón MÁ. 2007 Copro-necrophagous beetle (Coleoptera: Scarabaeidae) diversity in an agroecosystem in Yucatan, Mexico. *Rev. Biol. Trop.* **55**, 83–99.

169. Tind Nielsen S. 2007 Deforestation and biodiversity: effects of bushland cultivation on dung beetles in semi-arid Tanzania. *Biodivers. Conserv.* **16**, 2753–2769. (doi:10.1007/s10531-007-9213-3)

170. Escobar F, Halffter G, Arellano L. 2007 From forest to pasture: an evaluation of the influence of environment and biogeography on the structure of beetle (Scarabaeinae) assemblages along three altitudinal gradients in the Neotropical region. *Ecography (Cop.).* **30**, 193–208. (doi:10.1111/j.2007.0906-7590.04818.x)

171. Verdú JR, Moreno CE, Sánchez-Rojas G, Numa C, Galante E, Halffter G. 2007 Grazing promotes dung beetle diversity in the xeric landscape of a Mexican Biosphere Reserve. *Biol. Conserv.* **140**, 308–317. (doi:10.1016/j.biocon.2007.08.015)

172. Gardner TA, Hernández MIM, Barlow J, Peres CA. 2007 Understanding the biodiversity consequences of habitat change: the value of secondary and plantation forests for neotropical dung beetles. *J. Appl. Ecol.* **45**, 883–893. (doi:10.1111/j.1365-2664.2008.01454.x)

173. Horgan F. 2006 Aggregation and coexistence of dung beetles in montane rain forest and deforested sites in central Peru. *J. Trop. Ecol.* **22**, 359. (doi:10.1017/S026646740600321X)

174. Escobar F, Lobo JM, Halffter G. 2006 Assessing the origin of Neotropical mountain dung beetle assemblages (Scarabaeidae: Scarabaeinae): the comparative influence of vertical and horizontal colonization. *J. Biogeogr.* **33**, 1793–1803. (doi:10.1111/j.1365-2699.2006.01528.x)

175. Cabrero-Sanudo FJ, Lobo JM. 2006 Determinant variables of Iberian Peninsula Aphodiinae diversity (Coleoptera, Scarabaeoidea, Aphodiidae). *J. Biogeogr.* **33**, 1021–1043. (doi:10.1111/j.1365-2699.2006.01485.x)

176. Botes A, McGeoch MA, van Rensburg BJ. 2006 Elephant- and human-induced changes to dung beetle (Coleoptera: Scarabaeidae) assemblages in the Maputaland Centre of Endemism. *Biol. Conserv.* **130**, 573–583. (doi:10.1016/j.biocon.2006.01.020)

177. Vulinec K, Lambert JE, Mellow DJ. 2006 Primate and Dung Beetle Communities in Secondary Growth Rain Forests: Implications for Conservation of Seed Dispersal Systems. *Int. J. Primatol.* **27**, 855–879. (doi:10.1007/s10764-006-9027-2)

178. Davis AL, Scholtz C. 2005 A dung beetle survey of selected Gauteng nature reserves: implications for conservation of the provincial scarabaeine fauna. *African Entomol.* **13**, 1–16.

179. Escobar F, Lobo JM, Halffter G. 2005 Altitudinal variation of dung beetle (Scarabaeidae: Scarabaeinae) assemblages in the Colombian Andes. *Glob. Ecol. Biogeogr.* **14**, 327–337. (doi:10.1111/j.1466-822X.2005.00161.x)

180. Shahabuddin, Schulze CH, Tscharntke T. 2005 Changes of dung beetle communities from rainforests towards agroforestry systems and annual cultures in Sulawesi (Indonesia). *Biodivers. Conserv.* **14**, 863–877. (doi:10.1007/s10531-004-0654-7)

181. Arellano L, Favila ME, Huerta C. 2005 Diversity of dung and carrion beetles in a disturbed Mexican tropical montane cloud forest and on shade coffee plantations. *Biodivers. Conserv.* **14**, 601–615. (doi:10.1007/s10531-004-3918-3)

182. Durães R, Martins WP, Vaz-de-Mello FZ. 2005 Dung beetle (Coleoptera: Scarabaeidae) assemblages across a natural forest-cerrado ecotone in Minas Gerais, Brazil. *Neotrop. Entomol.* **34**, 721–731. (doi:10.1590/S1519-566X2005000500003)

183. Scheffler PY. 2005 Dung beetle (Coleoptera: Scarabaeidae) diversity and community structure across three disturbance regimes in eastern Amazonia. *J. Trop. Ecol.* **21**, 9–19. (doi:10.1017/S0266467404001683)

184. Avendaño-mendoza C, Morón-ríos A, Cano EB, León-Cortés J. 2005 Dung beetle community (Coleoptera: Scarabaeidae: Scarabaeinae) in a tropical landscape at the Lachua Region, Guatemala. *Biodivers. Conserv.* **14**, 801–822. (doi:10.1007/s10531-004-0651-x)

185. Horgan F. 2005 Effects of deforestation on diversity, biomass and function of dung beetles on the eastern slopes of the Peruvian Andes. *For. Ecol. Manage.* **216**, 117–133. (doi:10.1016/j.foreco.2005.05.049)

186. Andresen E. 2005 Effects of Season and Vegetation Type on Community Organization of Dung Beetles in a Tropical Dry Forest. *Biotropica* **37**, 291–300. (doi:10.1111/j.1744-7429.2005.00039.x)

187. Pineda E, Moreno C, Escobar F, Halffter G. 2005 Frog, Bat, and Dung Beetle Diversity in the Cloud Forest and Coffee Agroecosystems of Veracruz, Mexico. *Conserv. Biol.* **19**, 400–410. (doi:10.1111/j.1523-1739.2005.00531.x)

188. Quintero I, Roslin T. 2005 Rapid recovery of dung beetle communities following habitat fragmentation in Central Amazonia. *Ecology* **86**, 3303–3311. (doi:10.1890/04-1960)

189. Bertone M, Green J, Washburn S. 2005 Seasonal activity and species composition of dung beetles (Coleoptera: Scarabaeidae and Geotrupidae) inhabiting cattle pastures in North Carolina. *Ann. Entomol. Soc. Am.* **98**, 309–321. (doi:10.1603/0013-8746(2005)098%5B0309:SAASCO%5D2.0.CO%3B2)

190. Vernes K, Pope LC, Hill CJ, Bärlocher F. 2005 Seasonality, dung specificity and competition in dung beetle assemblages in the Australian Wet Tropics, north-eastern Australia. *J. Trop. Ecol.* **21**, 1–8. (doi:10.1017/S026646740400224X)

191. Kanda N, Yokota T, Shibata E, Sato H. 2005 Diversity of dung-beetle community in declining Japanese subalpine forest caused by an increasing sika deer population. *Ecol. Res.* **20**, 135–141. (doi:10.1007/s11284-004-0033-6)

192. Errouissi F, Jay-Robert P, Lumaret J-P, Piau O. 2004 Composition and Structure of Dung Beetle (Coleoptera: Aphodiidae, Geotrupidae, Scarabaeidae) Assemblages in Mountain Grasslands of the Southern Alps. *Ann. Entomol. Soc. Am.* **97**, 701–709. (doi:10.1603/0013-8746(2004)097[0701:CASODB]2.0.CO;2)

193. Escobar F. 2004 Diversity and composition of dung beetle (Scarabaeinae) assemblages in a heterogeneous Andean landscape. *Trop. Zool.* **17**, 123–136. (doi:10.1080/03946975.2004.10531202)

194. Davis AL, Scholtz CH. 2004 Local and regional species ranges of a dung beetle assemblage from the semi-arid Karoo/Kalahari margins, South Africa. *J. Arid Environ.* **57**, 61–85. (doi:10.1016/S0140-1963(03)00094-6)

195. Menéndez R, Gutiérrez D. 2004 Shifts in habitat associations of dung beetles in northern Spain: Climate change implications. *Écoscience* **11**, 329–337. (doi:10.1080/11956860.2004.11682840)

196. Andresen E. 2003 Effect of forest fragmentation on dung beetle communities and functional consequences for plant regeneration. *Ecography (Cop.).* **26**, 87–97. (doi:10.1034/j.1600-0587.2003.03362.x)

197. Spector S, Ayzama S. 2003 Rapid Turnover and Edge Effects in Dung Beetle Assemblages (Scarabaeidae ) at a Bolivian Neotropical Forest – Savanna Ecotone. *Biotropica* **35**, 394–404. (doi:10.1646/02102)

198. Krell FT, Krell-Westerwalbesloh S, Weiß I, Eggleton P, Linsenmair KE. 2003 Spatial separation of Afrotropical dung beetle guilds: A trade-off between competitive superiority and energetic constraints (Coleoptera: Scarabaeidae). *Ecography (Cop.).* **26**, 210–222. (doi:10.1034/j.1600-0587.2003.03278.x)

199. Hutton SA, Giller PS. 2003 The effects of the intensification of agriculture on northern temperate dung beetle communities. *J. Appl. Ecol.* **40**, 994–1007. (doi:10.1111/j.1365-2664.2003.00863.x)

200. Vessby K, Wiktelius S. 2003 The influence of slope aspect and soil type on immigration and emergence of some northern temperate dung beetles. *Pedobiologia (Jena).* **47**, 39–51. (doi:10.1078/0031-4056-00168)

201. Verdu JR, Galante E. 2002 Climatic stress, food availability and human activity as determinants of endemism patterns in the Mediterranean region: the case of dung beetles (Coleoptera, Scarabaeoidea) in the Iberian Peninsula. *Divers. Distrib.* **8**, 259–274. (doi:10.1046/j.1472-4642.2002.00151.x)

202. Medina C, Escobar F. 2002 Diversity and habitat use of dung beetles in a restored Andean landscape. *Biotropica* **34**, 181–187.

203. Vulinec K. 2002 Dung Beetle Communities and Seed Dispersal in Primary Forest and Disturbed Land in Amazonia. *Biotropica* **34**, 297–309. (doi:10.1111/j.1744-7429.2002.tb00541.x)

204. Estrada A, Coates-Estrada R. 2002 Dung beetles in continuous forest, forest fragments and in an agricultural mosaic habitat island at Los Tuxtlas, Mexico. *Biodivers. Conserv.* **11**, 1903–1918. (doi:10.1023/A:1020896928578)

205. Vessby K, Söderström B, Glimskär A, Svensson B. 2002 Species-Richness Correlations of Six Different Taxa in Swedish Seminatural Grasslands. *Conserv. Biol.* **16**, 430–439. (doi:10.1046/j.1523-1739.2002.00198.x)

206. Roslin T, Koivunen A. 2001 Distribution and abundance of dung beetles in fragmented landscapes. *Oecologia* **127**, 69–77. (doi:10.1007/s004420000565)

207. Jankielsohn A, Scholtz CH, Louw SV. 2001 Effect of Habitat Transformation on Dung Beetle Assemblages - A Comparison Between a South African Nature Reserve and Neighboring Farms. *Environ. Entomol.* **30**, 474–483. (doi:10.1603/0046-225X-30.3.474)

208. Davis AJ. 2000 Does Reduced-Impact Logging Help Preserve Biodiversity in Tropical Rainforests? A Case Study from Borneo using Dung Beetles (Coleoptera: Scarabaeoidea) as Indicators. *Environ. Entomol.* **29**, 467–475. (doi:10.1603/0046-225X-29.3.467)

209. Hortal-Muñoz J, Martín-Piera F, Lobo JM. 2000 Dung Beetle Geographic Diversity Variation Along a Western Dung Beetle Geographic Diversity Variation Along a Western Iberian Latitudinal Transect (Coleoptera : Scarabaeidae). *Ecol. Popul. Biol.* **93**, 235–243. (doi:10.1603/0013-8746(2000)093[0235:DBGDVA])

210. Romero-Alcaraz E, Ávila JM. 2000 Effect of elevation and type of habitat on the abundance and diversity of scarabaeoid dung beetle (scarabaeoidea) assemblages in a mediterranean area from southern iberian peninsula. *Zool. Stud.* **39**, 351–359.

211. Davis AJ, Huijbregts H, Krikken J. 2000 The role of local and regional processes in shaping dung beetle communities in tropical forest plantations in Borneo. *Glob. Ecol. Biogeogr.* **9**, 281–292. (doi:10.1046/j.1365-2699.2000.00189.x)

212. Escobar F., de Ulloa P. 2000 Distribución espacial y temporal en un gradiente de sucesión de la fauna de coleópteros coprófagos (Scarabaeinae, Aphodiinae) en un bosque tropical montano, Nariño-Colombia. *Rev. Biol. Trop.* **48**, 961–975.

213. Van Rensburg BJ, McGeoch MA, Chown SL, Van Jaarsveld AS. 1999 Conservation of heterogeneity among dung beetles in the Maputaland Centre of Endemism, South Africa. *Biol. Conserv.* **88**, 145–153. (doi:10.1016/S0006-3207(98)00109-8)

214. Barbero E, Palestrini C, Rolando A. 1999 Dung beetle conservation: Effects of habitat and resource selection (Coleoptera: Scarabaeoidea). *J. Insect Conserv.* **3**, 75–84. (doi:10.1023/A:1009609826831)

215. Davis AL, Scholtz CH, Chown SL. 1999 Species turnover, community boundaries and biogeographical composition of dung beetle assemblages across an altitudinal gradient in South Africa. *J. Biogeogr.* **26**, 1039–1055. (doi:10.1046/j.1365-2699.1999.00335.x)

216. Micó E, Verdú JR, Galante E. 1998 Diversity of Dung Beetles in Mediterranean Wetlands and Bordering Brushwood. *Ann. Entomol. Soc. Am.* **91**, 298–302. (doi:10.1093/aesa/91.3.298)

217. Davis AL. 1997 Climatic and biogeographical associations of southern African dung beetles (Coleoptera: Scarabaeidae s. str.). *Afr. J. Ecol.* **35**, 10–38. (doi:10.1111/j.1365-2028.1997.051-89051.x)

218. Kadiri N, Lobo JM, Lumaret J-P. 1997 Conséquences de l’interaction entre préférences pour l’habitat et quantité de ressources trophiques sur les communautés d’insectes coprophages (Coleoptera: Scarabaeoidea). *Acta Oecologica* **18**, 107–119. (doi:10.1016/S1146-609X(97)80068-9)

219. Davis AL. 1996 Community organization of dung beetles (Coleoptera: Scarabaeidae): differences in body size and functional group structure between habitats. *Afr. J. Ecol.* **34**, 258–275. (doi:10.1111/j.1365-2028.1996.tb00621.x)

220. Steenkamp HE, Chown SL. 1996 Influence of dense stands of an exotic tree, Prosopis glandulosa Benson, on a savanna dung beetle (Coleoptera: Scarabaeinae) assemblage in southern Africa. *Biol. Conserv.* **78**, 305–311. (doi:10.1016/S0006-3207(96)00047-X)

221. Davis AL. 1994 Community organization in a South African, winter rainfall, dung beetle assemblage (Coleoptera: Scarabaeidae). *Acta Oecologica* **15**, 727–738.

222. Davis AL. 1994 Habitat fragmentation in southern Africa and distributional response patterns in five specialist or generalist dung beetle families (Coleoptera). *Afr. J. Ecol.* **32**, 192–207. (doi:10.1111/j.1365-2028.1994.tb00571.x)

223. Giller PS, Doube BM. 1994 Spatial and Temporal Co-Occurrence of Competitors in Southern African Dung Beetle Communities. *J. Anim. Ecol.* **63**, 629–643.

224. Doube BM, Macqueen A. 1991 Establishment of exotic dung beetles in Queensland: the role of habitat specificity. *Entomophaga* **36**, 353–360. (doi:10.1007/BF02377939)

225. Doube B, Wardhaugh K. 1991 Habitat associations and niche partitioning in an island dung beetle community. *Acta Oecologica* **12**, 451–459.

226. Edwards P. 1991 Seasonal variation in the dung of African grazing mammals and its consequences for coprophagous insects. *Funct. Ecol.* **5**, 617–628. (doi:10.2307/2389480)

**Mammal association studies:**

1. Nichols E, Peres CA, Hawes JE, Naeem S. 2016 Multitrophic diversity effects of network degradation. *Ecol. Evol.* **6**, 4936–4946. (doi:10.1002/ece3.2253)
2. Pryke JS, Roets F, Samways MJ. 2016 Wild Herbivore Grazing Enhances Insect Diversity over Livestock Grazing in an African Grassland System. *PLoS One* **11**, e0164198. (doi:10.1371/journal.pone.0164198)
3. Bogoni JA *et al.* 2016 Contributions of the mammal community, habitat structure, and spatial distance to dung beetle community structure. *Biodivers. Conserv.* **25**, 1661–1675. (doi:10.1007/s10531-016-1147-1)
4. Feer F, Boissier O. 2015 Variations in dung beetle assemblages across a gradient of hunting in a tropical forest. *Ecol. Indic.* **57**. (doi:10.1016/j.ecolind.2015.04.034)
5. Koike S, Soga M, Nemoto Y, Kozakai C. 2014 How are dung beetle species affected by deer population increases in a cool temperate forest ecosystem? *J. Zool.* **293**, 227–233. (doi:10.1111/jzo.12138)
6. Culot L, Bovy E, Zagury Vaz-de-Mello F, Guevara R, Galetti M. 2013 Selective defaunation affects dung beetle communities in continuous Atlantic rainforest. *Biol. Conserv.* **163**, 79–89. (doi:10.1016/j.biocon.2013.04.004)
7. Tshikae BP, Davis AL V., Scholtz CH. 2013 Dung beetle assemblage structure across the aridity and trophic resource gradient of the Botswana Kalahari: patterns and drivers at regional and local scales. *J. Insect Conserv.* **17**, 623–636. (doi:10.1007/s10841-013-9547-y)
8. Nichols E, Uriarte M, Peres CA, Louzada J, Braga RF, Schiffler G, Endo W, Spector SH. 2013 Human-Induced Trophic Cascades along the Fecal Detritus Pathway. *PLoS One* **8**. (doi:10.1371/journal.pone.0075819)
9. Viljanen H, Escobar F, Hanski I. 2010 Low local but high beta diversity of tropical forest dung beetles in Madagascar. *Glob. Ecol. Biogeogr.* **19**, 886–894. (doi:10.1111/j.1466-8238.2010.00552.x)
10. Andresen E, Laurance SGW. 2007 Possible Indirect Effects of Mammal Hunting on Dung Beetle Assemblages in Panama. *Biotropica* **39**, 141–146. (doi:10.1111/j.1744-7429.2006.00239.x)
11. Lobo J. 2006 Regional and local influence of grazing activity on the diversity of a semi arid dung beetle community. *Divers. Distrib.* **12**, 111–123. (doi:10.1111/j.1366-9516.2006.00210.x)
12. Feer F, Hingrat Y. 2005 Effects of Forest Fragmentation on a Dung Beetle Community in French Guiana. *Conserv. Biol.* **19**, 1103–1112. (doi:10.1111/j.1523-1739.2005.00087.x)
13. Kanda N, Yokota T, Shibata E, Sato H. 2005 Diversity of dung-beetle community in declining Japanese subalpine forest caused by an increasing sika deer population. *Ecol. Res.* **20**, 135–141. (doi:10.1007/s11284-004-0033-6)
14. Verdu JR, Galante E. 2002 Climatic stress, food availability and human activity as determinants of endemism patterns in the Mediterranean region: the case of dung beetles (Coleoptera, Scarabaeoidea) in the Iberian Peninsula. *Divers. Distrib.* **8**, 259–274. (doi:10.1046/j.1472-4642.2002.00151.x)
15. Estrada A, Anzures D. A, Coates-Estrada R. 1999 Tropical rain forest fragmentation, howler monkeys (*Alouatta palliata*), and dung beetles at Los Tuxtlas, Mexico. *Am. J. Primatol.* **48**, 253–262.
16. Tonelli M, Verdú JR, Zunino M. 2018 Effects of the progressive abandonment of grazing on dung beetle biodiversity: body size matters. *Biodivers. Conserv.* **27**, 189–204. (doi:10.1007/s10531-017-1428-3)
17. Enari H, Koike S, Enari HS, Seki Y, Okuda K, Kodera Y. 2018 Early-stage ecological influences of population recovery of large mammals on dung beetle assemblages in heavy snow areas. *Acta Oecologica* **92**, 7–15. (doi:10.1016/j.actao.2018.07.007)
18. Raine EH, Mikich SB, Lewis OT, Riordan P, Vaz-de-Mello FZ, Slade EM. 2018 Extinctions of interactions: quantifying a dung beetle--mammal network. *Ecosphere* **9**, e02491. (doi:10.1002/ecs2.2491)
19. Iida T, Soga M, Koike S. 2018 Large herbivores affect forest ecosystem functions by altering the structure of dung beetle communities. *Acta Oecologica* **88**, 65–70. (doi:10.1016/j.actao.2018.03.003)
20. Alvarado F, Escobar F, Williams DR, Arroyo-Rodríguez V, Escobar-Hernández F. 2018 The role of livestock intensification and landscape structure in maintaining tropical biodiversity. *J. Appl. Ecol.* **55**, 185–194. (doi:10.1111/1365-2664.12957)
21. Barlow J, Louzada J, Parry L, Hernández MIM, Hawes J, Peres CA, Vaz-de-Mello FZ, Gardner TA. 2010 Improving the design and management of forest strips in human-dominated tropical landscapes: A field test on Amazonian dung beetles. *J. Appl. Ecol.* **47**, 779–788. (doi:10.1111/j.1365-2664.2010.01825.x)

**Ecosystem functioning references:**

1. Santos-Heredia C, Andresen E, Zárate DA, Escobar F. 2018 Dung beetles and their ecological functions in three agroforestry systems in the Lacandona rainforest of Mexico. *Biodivers. Conserv.* **27**, 2379–2394. (doi:10.1007/s10531-018-1542-x)

2. Culot L, Huynen MC, Heymann EW. 2018 Primates and Dung Beetles: Two Dispersers Are Better than One in Secondary Forest. *Int. J. Primatol.* **39**, 397–414. (doi:10.1007/s10764-018-0041-y)

3. Piccini I, Nervo B, Forshage M, Celi L, Palestrini C, Rolando A, Roslin T. 2018 Dung beetles as drivers of ecosystem multifunctionality: Are response and effect traits interwoven? *Sci. Total Environ.* **616**–**617**, 1440–1448. (doi:10.1016/j.scitotenv.2017.10.171)

4. Badenhorst J, Dabrowski J, Scholtz CH, Truter WF. 2018 Dung beetle activity improves herbaceous plant growth and soil properties on confinements simulating reclaimed mined land in South Africa. *Appl. Soil Ecol.* **132**, 53–59. (doi:10.1016/j.apsoil.2018.08.011)

5. Forgie SA, Paynter Q, Zhao Z, Flowers C, Fowler S V. 2018 Newly released non-native dung beetle species provide enhanced ecosystem services in New Zealand pastures. *Ecol. Entomol.* **43**, 431–439. (doi:10.1111/een.12513)

6. Carvalho RL, Frazão F, Ferreira-Châline RS, Louzada J, Cordeiro L, França F. 2018 Dung burial by roller dung beetles (Coleoptera: Scarabaeinae): An individual and specific-level study. *Int. J. Trop. Insect Sci.* **38**, 373–380. (doi:10.1017/S1742758418000206)

7. Nunes CA, Braga RF, de Moura Resende F, de Siqueira Neves F, Figueira JEC, Fernandes GW. 2018 Linking Biodiversity, the Environment and Ecosystem Functioning: Ecological Functions of Dung Beetles Along a Tropical Elevational Gradient. *Ecosystems* **21**, 1244–1254. (doi:10.1007/s10021-017-0216-y)

8. Iida T, Soga M, Koike S. 2018 Large herbivores affect forest ecosystem functions by altering the structure of dung beetle communities. *Acta Oecologica* **88**, 65–70. (doi:10.1016/j.actao.2018.03.003)

9. González-Tokman D, Cultid-Medina C, Díaz A, Escobar F, Ocampo-Palacio L, Martínez-Garza C. 2018 Success or failure: the role of ecological restoration on the recovery of dung beetle diversity and function in a tropical rainforest. *Rev. Mex. Biodivers.* **89**, 232–242. (doi:10.22201/ib.20078706e.2018.1.2132)

10. França F, Louzada J, Barlow J. 2018 Selective logging effects on ‘brown world’ faecal-detritus pathway in tropical forests: A case study from Amazonia using dung beetles. *For. Ecol. Manage.* **410**, 136–143. (doi:10.1016/j.foreco.2017.12.027)

11. Manning P, Cutler GC. 2018 Ecosystem functioning is more strongly impaired by reducing dung beetle abundance than by reducing species richness. *Agric. Ecosyst. Environ.* **264**, 9–14. (doi:10.1016/j.agee.2018.05.002)

12. Amore V, da Silva PG, Hensen MC, Hernández MIM, Lobo JM. 2018 Variation in dung removal by dung beetles in subtropical Atlantic Rainforests. *Entomol. Exp. Appl.* **166**, 854–862. (doi:10.1111/eea.12724)

13. Frank K, Hülsmann M, Assmann T, Schmitt T, Blüthgen N. 2017 Land use affects dung beetle communities and their ecosystem service in forests and grasslands. *Agric. Ecosyst. Environ.* **243**, 114–122. (doi:10.1016/j.agee.2017.04.010)

14. Batilani-Filho M, Hernandez MIM. 2017 Decline of Ecological Functions Performed by Dung Beetles in Areas of Atlantic Forest and Contribution of Rollers and Tunnellers in Organic Matter Removal. *Environ. Entomol.* **46**, 784–793. (doi:10.1093/ee/nvx091)

15. Nervo B *et al.* 2017 Ecological functions provided by dung beetles are interlinked across space and time: Evidence from 15N isotope tracing. *Ecology* **98**, 433–446. (doi:10.1002/ecy.1653)

16. Slade EM, Kirwan L, Bell T, Philipson CD, Lewis OT, Roslin T. 2017 The importance of species identity and interactions for multifunctionality depends on how ecosystem functions are valued. *Ecology* **98**, 2626–2639. (doi:10.1002/ecy.1954)

17. Milotić T, Quidé S, Van Loo T, Hoffmann M. 2017 Linking functional group richness and ecosystem functions of dung beetles: an experimental quantification. *Oecologia* **183**, 177–190. (doi:10.1007/s00442-016-3756-5)

18. Lugon AP, Boutefeu M, Bovy E, Vaz-de-Mello FZ, Huynen M, Galetti M, Culot L. 2017 Persistence of the effect of frugivore identity on post-dispersal seed fate: consequences for the assessment of functional redundancy. *Biotropica* **49**, 293–302. (doi:10.1111/btp.12418)

19. França FM, Frazão FS, Korasaki V, Louzada J, Barlow J. 2017 Identifying thresholds of logging intensity on dung beetle communities to improve the sustainable management of Amazonian tropical forests. *Biol. Conserv.* **216**, 115–122. (doi:10.1016/j.biocon.2017.10.014)

20. De Farias PM, Hernández MIM. 2017 Dung beetles associated with agroecosystems of southern Brazil: Relationship with soil properties. *Rev. Bras. Cienc. do Solo* **41**, 1–13. (doi:10.1590/18069657rbcs20160248)

21. Braga RF, Carvalho R, Andresen E, Anjos DV., Alves-Silva E, Louzada J. 2017 Quantification of four different post-dispersal seed deposition patterns after dung beetle activity. *J. Trop. Ecol.* **33**, 407–410. (doi:10.1017/S0266467417000335)

22. Piccini I, Arnieri F, Caprio E, Nervo B, Pelissetti S, Palestrini C, Roslin T, Rolando A. 2017 Greenhouse gas emissions from dung pats vary with dung beetle species and with assemblage composition. *PLoS One* **12**, e0178077. (doi:10.1371/journal.pone.0178077)

23. Santos-Heredia C, Andresen E, Del-Val E, Zárate DA, Nava Mendoza M, Jaramillo VJ. 2016 The activity of dung beetles increases foliar nutrient concentration in tropical seedlings. *Biotropica* **48**, 565–567. (doi:10.1111/btp.12364)

24. Slade EM, Riutta T, Roslin T, Tuomisto HL. 2016 The role of dung beetles in reducing greenhouse gas emissions from cattle farming. *Sci. Rep.* **6**, 1–9. (doi:10.1038/srep18140)

25. Ortega-Martínez IJ, Moreno CE, Escobar F. 2016 A dirty job: manure removal by dung beetles in both a cattle ranch and laboratory setting. *Entomol. Exp. Appl.* **161**, 70–78. (doi:10.1111/eea.12488)

26. Derhé MA, Murphy H, Monteith G, Menéndez R. 2016 Measuring the success of reforestation for restoring biodiversity and ecosystem functioning. *J. Appl. Ecol.* **53**, 1714–1724. (doi:10.1111/1365-2664.12728)

27. Kenyon TM, Mayfield MM, Monteith GB, Menéndez R. 2016 The effects of land use change on native dung beetle diversity and function in Australia’s Wet Tropics. *Austral Ecol.* **41**, 797–808. (doi:10.1111/aec.12366)

28. Slade EM, Roslin T, Santalahti M, Bell T. 2016 Disentangling the ‘brown world’ faecal-detritus interaction web: dung beetle effects on soil microbial properties. *Oikos* **125**, 629–635. (doi:10.1111/oik.02640)

29. Johnson SN, Lopaticki G, Barnett K, Facey SL, Powell JR, Hartley SE. 2016 An insect ecosystem engineer alleviates drought stress in plants without increasing plant susceptibility to an above-ground herbivore. *Funct. Ecol.* **30**, 894–902. (doi:10.1111/1365-2435.12582)

30. Menéndez R, Webb P, Orwin KH. 2016 Complementarity of dung beetle species with different functional behaviours influence dung–soil carbon cycling. *Soil Biol. Biochem.* **92**, 142–148. (doi:10.1016/j.soilbio.2015.10.004)

31. Ardali EO, Tahmasebi P, Bonte D. 2016 Ecological Sustainability in Rangelands: The Contribution of Dung Beetles in Secondary Seed Dispersal (Case study: Chaharmahal and Bakhtiari province, Iran). *Eur. J. Sustain. Dev.* **6**, 133–139. (doi:10.14207/ejsd.2016.v5n3p133)

32. Griffiths HM, Bardgett RD, Louzada J, Barlow J. 2016 The value of trophic interactions for ecosystem function: dung beetle communities influence seed burial and seedling recruitment in tropical forests. *Proc. R. Soc. B Biol. Sci.* **283**, 20161634. (doi:10.1098/rspb.2016.1634)

33. Manning P, Slade EM, Beynon SA, Lewis OT. 2016 Functionally rich dung beetle assemblages are required to provide multiple ecosystem services. *Agric. Ecosyst. Environ.* **218**, 87–94. (doi:10.1016/j.agee.2015.11.007)

34. Slade EM, Roslin T. 2016 Dung beetle species interactions and multifunctionality are affected by an experimentally warmed climate. *Oikos* **125**, 1607–1616. (doi:10.1111/oik.03207)

35. Tixier T, Bloor JMG, Lumaret JP. 2015 Species-specific effects of dung beetle abundance on dung removal and leaf litter decomposition. *Acta Oecologica* **69**, 31–34. (doi:10.1016/j.actao.2015.08.003)

36. Griffiths HM, Louzada J, Bardgett RD, Beiroz W, França F, Tregidgo D, Barlow J. 2015 Biodiversity and environmental context predict dung beetle-mediated seed dispersal in a tropical forest field experiment. *Ecology* **96**, 1607–1619. (doi:10.1890/14-1211.1)

37. Iwasa M, Moki Y, Takahashi J. 2015 Effects of the activity of coprophagous insects on greenhouse gas emissions from cattle dung pats and changes in amounts of nitrogen, carbon, and energy. *Environ. Entomol.* **44**, 106–113. (doi:10.1093/ee/nvu023)

38. Yoshihara Y, Sato S. 2015 The relationship between dung beetle species richness and ecosystem functioning. *Appl. Soil Ecol.* **88**, 21–25. (doi:10.1016/j.apsoil.2014.12.001)

39. Nervo B, Tocco C, Caprio E, Palestrini C, Rolando A. 2014 The Effects of Body Mass on Dung Removal Efficiency in Dung Beetles. *PLoS One* **9**, e107699. (doi:10.1371/journal.pone.0107699)

40. Enari H, Sakamaki-Enari H. 2014 Synergistic effects of primates and dung beetles on soil seed accumulation in snow regions. *Ecol. Res.* **29**, 653–660. (doi:10.1007/s11284-014-1152-3)

41. Santos-Heredia C, Andresen E. 2014 Upward movement of buried seeds: Another ecological role of dung beetles promoting seedling establishment. *J. Trop. Ecol.* **30**, 409–417. (doi:10.1017/S0266467414000376)

42. Braga RF, Korasaki V, Andresen E, Louzada J. 2013 Dung Beetle Community and Functions along a Habitat-Disturbance Gradient in the Amazon: A Rapid Assessment of Ecological Functions Associated to Biodiversity. *PLoS One* **8**, e57786. (doi:10.1371/journal.pone.0057786)

43. Penttilä A, Slade EM, Simojoki A, Riutta T, Minkkinen K, Roslin T. 2013 Quantifying Beetle-Mediated Effects on Gas Fluxes from Dung Pats. *PLoS One* **8**, e71454. (doi:10.1371/journal.pone.0071454)

44. Gollan JR, de Bruyn LL, Reid N, Wilkie L. 2013 Monitoring the ecosystem service provided by dung beetles offers benefits over commonly used biodiversity metrics and a traditional trapping method. *J. Nat. Conserv.* **21**, 183–188. (doi:10.1016/j.jnc.2012.12.004)

45. Feer F, Ponge J-F, Jouard S, Gomez D. 2013 Monkey and dung beetle activities influence soil seed bank structure. *Ecol. Res.* **28**, 93–102. (doi:10.1007/s11284-012-1006-9)

46. Kudavidanage EP, Qie L, Lee JSH. 2012 Linking Biodiversity and Ecosystem Functioning of Dung Beetles in South and Southeast Asian Tropical Rainforests. *Raffles Bull. Zool.* 25, 141–154.

47. Dangles O, Carpio C, Woodward G. 2012 Size-dependent species removal impairs ecosystem functioning in a large-scale tropical field experiment. *Ecology* **93**, 2615–2625. (doi:10.1890/12-0510.1)

48. Braga RF, Korasaki V, Audino LD, Louzada J. 2012 Are Dung Beetles Driving Dung-Fly Abundance in Traditional Agricultural Areas in the Amazon? *Ecosystems* **15**, 1173–1181. (doi:10.1007/s10021-012-9576-5)

49. Lawson CR, Mann DJ, Lewis OT. 2012 Dung Beetles Reduce Clustering of Tropical Tree Seedlings. *Biotropica* **44**, 271–275. (doi:10.1111/j.1744-7429.2012.00871.x)

50. Slade EM, Mann DJ, Lewis OT. 2011 Biodiversity and ecosystem function of tropical forest dung beetles under contrasting logging regimes. *Biol. Conserv.* **144**, 166–174. (doi:10.1016/j.biocon.2010.08.011)

51. GIRALDO C, ESCOBAR F, CHARÁ JD, CALLE Z. 2011 The adoption of silvopastoral systems promotes the recovery of ecological processes regulated by dung beetles in the Colombian Andes. *Insect Conserv. Divers.* **4**, 115–122. (doi:10.1111/j.1752-4598.2010.00112.x)

52. Santos-Heredia C, Andresen E, Stevenson P. 2011 Secondary seed dispersal by dung beetles in an Amazonian forest fragment of Colombia: Influence of dung type and edge effect. *Integr. Zool.* **6**, 399–408. (doi:10.1111/j.1749-4877.2011.00261.x)

53. Culot L, Mann DJD, Lazo FJJMFM, Huynen M, Heymann EW. 2011 Tamarins and dung beetles: An efficient diplochorous dispersal system in the Peruvian Amazonia. *Biotropica* **43**, 84–92. (doi:10.1111/j.1744-7429.2010.00655.x)

54. Kunz BK, Krell F-TT. 2011 Habitat differences in dung beetle assemblages in an African savanna-forest ecotone: implications for secondary seed dispersal. *Integr. Zool.* **6**, 81–96. (doi:10.1111/j.1749-4877.2011.00240.x)

55. Santos-Heredia C, Andresen E, Zárate DA. 2010 Secondary seed dispersal by dung beetles in a Colombian rain forest: effects of dung type and defecation pattern on seed fate. *J. Trop. Ecol.* **26**, 355–364. (doi:10.1017/S0266467410000192)

56. Amézquita S, Favila ME. 2010 Removal Rates of Native and Exotic Dung by Dung Beetles (Scarabaeidae: Scarabaeinae) in a Fragmented Tropical Rain Forest. *Environ. Entomol.* **39**, 328–336. (doi:10.1603/EN09182)

57. Brown J, Scholtz CH, Janeau J-L, Grellier S, Podwojewski P. 2010 Dung beetles (Coleoptera: Scarabaeidae) can improve soil hydrological properties. *Appl. Soil Ecol.* **46**, 9–16. (doi:10.1016/j.apsoil.2010.05.010)

58. Lee JSH, Lee IQW, Lim SL-H, Huijbregts J, Sodhi NS. 2009 Changes in dung beetle communities along a gradient of tropical forest disturbance in South-East Asia. *J. Trop. Ecol.* **25**, 677–680. (doi:10.1017/S0266467409990174)

59. Rosenlew H, Roslin T. 2008 Habitat fragmentation and the functional efficiency of temperate dung beetles. *Oikos* **117**, 1659–1666. (doi:10.1111/j.1600-0706.2008.16904.x)

60. Slade EM, Mann DJ, Villanueva JF, Lewis OT. 2007 Experimental evidence for the effects of dung beetle functional group richness and composition on ecosystem function in a tropical forest. *J. Anim. Ecol.* **76**, 1094–1104. (doi:10.1111/j.1365-2656.2007.01296.x)

61. Bang HS, Lee JH, Oh SK, Young EN, Yong SJ, Won HK. 2005 Effects of paracoprid dung beetles (Coleoptera: Scarabaeidae) on the growth of pasture herbage and on the underlying soil. *Appl. Soil Ecol.* **29**, 165–171. (doi:10.1016/j.apsoil.2004.11.001)

62. Horgan F. 2005 Effects of deforestation on diversity, biomass and function of dung beetles on the eastern slopes of the Peruvian Andes. *For. Ecol. Manage.* **216**, 117–133. (doi:10.1016/j.foreco.2005.05.049)

63. Bustamante-Sánchez MA, Grez AA, Simonetti JA. 2004 Dung decomposition and associated beetles in a fragmented temperate forest. *Rev. Chil. Hist. Nat.* **77**, 107–120. (doi:10.4067/S0716-078X2004000100009)

64. Chapman CA, Chapman LJ, Vulinec K, Zanne A, Lawes MJ. 2003 Fragmentation and Alteration of Seed Dispersal Processes: An Initial Evaluation of Dung Beetles, Seed Fate, and Seedling Diversity1. *Biotropica* **35**, 382–393. (doi:10.1111/j.1744-7429.2003.tb00592.x)

65. Vulinec K. 2002 Dung Beetle Communities and Seed Dispersal in Primary Forest and Disturbed Land in Amazonia1. *Biotropica* **34**, 297–309. (doi:10.1111/j.1744-7429.2002.tb00541.x)

66. Andresen E. 2001 Effects of dung presence, dung amount and secondary dispersal by dung beetles on the fate of *Micropholis guyanensis* (Sapotaceae) seeds in Central Amazonia. *J. Trop. Ecol.* **17**, 61–78. (doi:10.1017/S0266467401001043)

67. Feer F. 1999 Effects of dung beetles (Scarabaeidae) on seeds dispersed by howler monkeys (*Alouatta seniculus*) in the French Guianan rain forest. *J. Trop. Ecol.* **15**, 129–142. (doi:10.1017/S0266467499000711)

68. Yokoyama K, Kai H, Tsuchiyama H. 1991 Paracoprid dung beetles and gaseous loss of nitrogen from cow dung. *Soil Biol. Biochem.* **23**, 643–647. (doi:10.1016/0038-0717(91)90077-W)

**Table S1.** Studies that explore dung beetle - mammal associations using dung beetle and mammal co-occurrence data. Explanation of *Methods* classifications: *Mammal species abundance/density* (Raw abundance/density calculated from the total survey time carried out in each area and the number of sightings); *Mammal species richness* (presence/absence from mammal survey data); *Qualitative index* (categorical classifications of large mammal presence/density); *Human disturbance* (hunting or human disturbance as a proxy for mammal defaunation); *Focal species* (study of one or several species known a priori to contribute a significant proportion to the dung profile); *Grazing intensity* (as an estimate for mammal/dung density); *Literature* (deriving mammal species composition and density from IUCN lists or aerial surveys). *Small mammal data:* Inclusion of data for mammals <1.5kg. Direction of effect of mammals on dung beetles: if a decline in mammal abundance or richness led to a decline in dung beetle abundance or another metric, this is signified by ‘-’. If the dung beetle community composition varied significantly among areas with different mammal species composition, this is signified by ‘≠’. Non-significant differences are signified by ‘=’. Empty cells represent no data, or no analysis carried out.

| **Author** | **Methods** | | | | **Direction of effect of mammal decline on dung beetles:** | | | |
| --- | --- | --- | --- | --- | --- | --- | --- | --- |
|  | **Location** | **Mammal data classification** | **Small mammal data** | **Additional explanatory variables** | **Sp. richness** | **Abundance** | **Community composition** | **Additional findings** |
| Enari 2018 | Japan | Mammal abundance | Y |  |  |  | ≠ |  |
| Iida 2018 | Japan | Focal sp. | N | Understory cover  Soil hardness | - Rate of dung decomposition  - small bodied beetles | | | |
| Raine *et al.* 2018 | Atlantic Forest, Brazil | Mammal abundance | Y |  | Simulated mammal extinctions to predict effects on dung beetle population metrics | | | |
| Tonelli et al. 2018 | Italy | Grazing intensity | N |  | - | - | ≠ | - Dung beetle biomass - Large bodied beetles |
| Alvarado *et al.* 2018 | Mexico | Grazing intensity | N | Landscape structure |  |  | = | Dung beetle species richness, biomass and composition more strongly related to landscape structure than grazing intensity. |
| Nichols *et al.* 2016 | Brazil | Mammal spp. richness | Y |  | Simulated mammal extinctions to predict effects on dung beetle population metrics | | | |
| Bogoni *et al.* 2016 | Brazil | Mammal abundance Mammal spp. richness | Y | Habitat structure | - |  |  | 40% of dung beetle species composition attributed to mammal species composition |
| Pryke *et al.* 2016 | South Africa | Grazing intensity | N | Fragment size |  |  | ≠ | Quantity of dung explained 60% of variance in species richness |
| Feer & Boissier 2015 | French Guiana | Qualitative index  Hunting proxy | Y |  |  | = | ≠ | Beetle size varied between sites and with hunting pressure |
| Koike *et al.* 2014 | Japan | Focal sp. | N | Understory cover | - |  | ≠ |  |
| Culot *et al.* 2013 | Brazil | Mammal abundance  Mammal spp. richness | Y |  | - | + |  | + Small bodied beetles  - Large bodied beetles |
| Nichols *et al.* 2013 | Brazil | Mammal abundance  Hunting | Y |  | = |  | ≠ |  |
| Tshikae 2013 | Botswana | Literature | N | Spatial distance, aridity, vegetation, soil | Large mammal composition was the third strongest factor influencing regional patterns of dung beetle species assemblages. | | | |
| Barlow *et al.* 2010 | Brazil | Qualitative index | N | Forest structure, landscape context | = | - | = |  |
| Viljanen 2010 | Madagascar, C&S America, Africa, SE Asia | Literature | N |  | - |  |  |  |
| Andresen & Laurance 2007 | Panama | Mammal abundance  Mammal spp. richness  Hunting | N | Dry season days | - | - | ≠ | + Proportion of nocturnal beetles |
| Lobo 2006 | Spain | Focal sp.  Grazing intensity | Y |  | - | - |  |  |
| Kanda *et al.* 2005 | Japan | Focal sp. | N |  | - | - |  |  |
| Feer & Hingrat 2005 | French Guiana | Focal sp.  Mammal spp. richness | N |  | - |  | ≠ |  |
| Verdu 2002 | Iberian Peninsula | Grazing intensity | N | Bioclimatic factors |  |  |  | Rate of dung beetle endemism affected by faunal composition |
| Estrada 1999 | Mexico | Focal sp. | N | Season, forest type |  | - |  |  |

**Supplementary Table S2:** Dung beetle diet preference studies where at least two dung types were used to capture dung beetles in an area. *Additional factors*: factors included in the analysis in addition to variation in dung type. *Methods/trap spacing*: lab-based studies recorded as ‘lab’, field studies record the spacing between traps. *Dung volume*: ‘NS’: not stated in the study, ‘realistic’: collected from dung mounds found in the wild, dung volume not stated.

| **Paper** | **Ecosystem** | **Location** | **Additional factors** | **Methods/ trap spacing** | **Dung volume** | **No bait sources** | **Mammal dung used as bait** |
| --- | --- | --- | --- | --- | --- | --- | --- |
| Al-Houty & Al-Musalam 1997 | - | Kuwait |  | lab | Realistic | 5 | Horse, sheep, camel, Arabian fox, dog |
| Ameziquita & Favila 2010 | Tropical rain forest and cloud forest | Mexico | Fragment size, dung removal | 50m, linear | 200g | 2 | Spider monkey, cow |
| Barbero *et al.* 1999 | Mosaic of wooded and open patches | Italy |  | Blocks: traps>3 m apart | 300g | 4 | Cow, wild boar, horse, sheep |
| Bogoni & Hernandez 2014 | Atlantic forest | Brazil |  | 25m apart, block | 10g | 4 | Puma, Crab-eating fox, black-horned capuchin, tapir |
| Carpento *et al.* 2010 | Savanna, open grasslands | Uganda | Soil type | Realistic | Realistic | 3 | Hippo, buffalo, warthog |
| Carpento *et al.* 2005 | Small deciduous woodlands, grasslands and ponds, urban and suburban areas | Italy |  | Realistic | ~5L | 4 | Sheep, dog, cow, horse |
| Correa & Puker 2016 | Cerrado | Brazil | Habitat | Blocks 50m apart, traps  10m apart within block | 50ml | 4 | cattle, human, pig, (and carrion) |
| Davis *et al.*  2010 | Open grassland | South Africa |  | Blocks: traps 50m apart within block | 250g | 4 | Elephant, cow, sheep, pig |
| Dormont *et al*. 2004 | Mediterranean climate: Pasture vegetation and herbaceous pasture | France |  | Blocks 10m apart, with traps 1m apart within block & lab | 300g | 2 | horse, cattle |
| Dormont *et al.* 2007 |  | South Central France |  | Blocks 10m apart, 1m apart within block | 300g | 4 | Sheep, cattle, horse, red deer |
| Edwards 1991 | Hot summer-rainfall regions South Africa | South Africa | Dung water content | Lab | - | 3 | Zebra, wildebeest, impala |
| Errousi *et al.* 2004 | Bioclimatic gradient of pasture land | France, Morocco | Bait size | 10m linear | 30g, 750g | 3 | Cow, goat, sheep |
| Enari *et al.* 2013 | Cool temperate forest, beech and oak | Japan |  | 10m linear | 15g | 5 | Macaque, Asiatic black bear, Japanese serow, Temminck and cattle |
| Enari *et al.* 2016 | Cool temperate forest, beech and oak | Japan |  | 50m, linear | Realistic |  | Macaque, Japanese serow, Japanese hare |
| Estrada *et al.* 1993 | Lowland evergreen tropical rainforest | Mexico |  | Blocks 20m apart, traps 5m apart within block | 5g | 2 | Howler monkey and coati |
| Frank *et al.* 2017 | Forest, grassland | Germany | Land use | 10m linear | 35g | 6 | Cow, horse, deer, sheep, fox, wild boar |
| Frank *et al.* 2017 | Forest, grassland | Germany |  | Field | 35g | 12 | Wolf, lynx, fox, brown bear, wild boar, cow, horse, sheep, deer, elephant, elk and wisent |
| Frank *et al.* 2018 |  | Germany | Volatile organic compound composition | Lab | - | 6 | Cow, horse, sheep, red deer, wild boar, fox |
| Filgueiras  *et al.*2009 | Brazilian Atlantic rainforest fragment | Brazil |  | Traps 2m apart blocks 15m apart | NS | 3 | Human, jaguar and waterbuck |
| Finn & Giller 2002 | Cattle pasture, wildlife park | Ireland |  | 5m | 1000g | 3 | Cow, horse, sheep |
| Galante & Cartagena 1999 | Mediterranean forest | Spain |  | Realistic | 1000g | 2 | Rabbit and cow |
| Giménez Gómez *et al.* 2018 | Atlantic forest | Argentina | Land use | Traps 50m apart | NS | 5 | Ocelot, lowland tapir, brocket deer, black and gold howler monkey, hooded capuchin |
| Gittings & Giller 1998 | Grazed pastures | Ireland |  | Grid: 5m apart | 1l | 6 | Cow, giraffe, zebra, guanaco, ostrich, red lechwe |
| Hewavithana *et al.* 2016 | Tropical monsoon forest | Sri Lanka | Habitat | 50m, linear | 200ml | 5 | leopard, cervid, elephant, bear, buffalo |
| Jones *et al.* 2012 | - | New Zealand | Native/ exotic diet sources | lab | 4-6g | 18 | Dung: Cow, sheep, pig, brushtail possum, dog, kakapo, kereu, kiwi, emu, caterpillar, wetapunga, phasmid, tuatura. Also mushroom, rotting banana and purri fruit, carrion and humus. |
| Larsen *et al.* 2006 | Forest of varying disturbance | Peru | Extreme specialists | 50m, linear | NS | 28 | Dung: human, cow, horse, tayra, puma, lizard, bird and howler monkey and snail. Carrion: Lizard, chicken, fish, rodent, frogs, snakes and opossum. Insects: grasshoppers, beetles, millipedes, caterpillars and cockroaches. Rotting fruit: banana, kiwi, papaya, tomato, passion fruit, cacao and other unidentified fruits of the forest. |
| Marsh *et al.* 2013 | Primary rainforest, secondary forest and plantations | Brazil | Optimum bait | 100m, linear | 20g | 2 | Human, pig and mix |
| Martin-Piera & Lobo 1996 | Mediterranean forest | Spain |  | Grid: ~10m | 1000g, 250g | 8 | Cow, deer, horse, fox, lynx, human, badger and wild boar |
| Medina & Lopes 2014 | Seasonally dry tropical forest | Brazil |  | Blocks 2m apart 25 between | NS | 4 | Carrion (bovine spleen), rotten banana, human and cow |
| Mroczynski *et al.* 2014 | Grazed meadow | Poland |  | Grid: 30cm | Realistic | 2 | Cattle and horse |
| Noriega 2012 | Mixture lowland wet tropical forest | Colombia |  | Realistic | realistic | 2 | Wooly monkey and howler monkey |
| Plewinska 2007 | Mixed coniferous forest | Poland |  | 10m, linear | 21g | 5 | bank vole, horse, cow, yellow Gouda cheese, rodent |
| Puker *et al.* 2013 | Riparian strips, tropical forest | Brazil | Habitat | 20m between traps, 100m between groups | 40g | 2 | Human and capybara |
| Santos-Heredia *et al.* 2010 | Moist forest fragments, agricultural land | Colombia |  | 50m, linear | 30g | 2 | Spider monkey and howler monkey |
| Shahabudin *et al.* 2010 | Rainforest, agroforestry | Indonesia | Habitat disturbance | 10m, linear | 26g | 2 | Cattle dung and anoa dung (small buffalo) |
| Siddall 2004 | Evergreen forest | Uganda |  | Blocks, traps 10m apart and 200m between blocks |  | 2 | Primate and elephant |
| Sites *et al.* 2018 | Seasonal evergreen forest | Thailand |  | 20m spacing | 8cm^3^ | 4 | Pigtail macaque, barking deer, sambar deer, Asian elephant |
| Stavert *et al.* 2014 | Waitakere Ranges | New Zealand | Native/ exotic diet sources | Lab | 3-5g | 22 | Dung: tuatara, short tailed bat, Kakapo, Brown kiwi, Australasian gannet, Brown teal, Weka. Mammal dung: Cow, Pig, Dog, Common brushtail possum, sheep, horse. Insect dung: Wetapunga, green looper caterpillar, stick insect. Carrion: earthwork, mealwork, locust, chicken, beef, squid |
| Tshikae *et al.* 2008 | Woodland | Botswana | Dung source | 50m linear | 250g | 5 | Pig, cattle, sheep, elephant dung and chicken livers |
| Tshikae *et al.* 2013 | Desert | Botswana | Aridity gradient, dung source | 50m linear | 250g | 5 | Pig, cattle, sheet, elephant dung and chicken livers |
| Vernes *et al.* 2005 | Eucalyptus woodland, allocasuarina forest | Australia |  | 100m, linear | Realistic | 5 | Northern Brown Bandicoot, Northern Bettong, Giant white tailed rat, norther quoll, long nosed bandicoot |
| Vinod & Sabu 2007 | Moist forests | India |  | 100m, linear | 1000cm3 | 2 | Gaur and Asian Elephant |
| Whipple & Holback 2012 | Pasture | US | Native/exotic diet sources | 100m linear | 113g | 15 | American bison, Shiras moose, cougar, pig, human, chimp, Bengal tiger, Africa lion, zebra, waterbuck and carrion. |
| Wurmitzer *et al.*  2017 | Lowland monte desert and meadow | Argentina, Austria | Real/ synthetic odours Habitat | 20m, linear | 15-30g | 5, 3 | Argentina: Horse, buffalo, capybara, patagonian mara, guanaco. Austria: horse, donkey and cow. |

**Supplementary Table S3:** Ecosystem functions studied in dung beetle diet preference papers. EF no.: number of ecosystem functions used to trap dung beetles. Bait no: number of bait types used to trap dung beetles.

| **Paper** | **EF no.** | **Ecosystem functions** | **Dung type** | **Bait Size (g)** | **Bait no.** |
| --- | --- | --- | --- | --- | --- |
| Culot *et al.* 2018 | 3 | Seed burial, seed dispersal, germination | Saddleback tamarin, Moustached tamarin | Natural | 2 |
| Piccini *et al.* 2018 | 3 | Dung removal, soil nutrients, greenhouse gas emission | Cattle | 1000 | 1 |
| Badenhorst *et al.* 2018 | 4 | Plant growth, soil properties, soil nutrients, soil bioturbation | Cattle | 1000 | 1 |
| Forgie *et al.* 2018 | 2 | Soil hydrological properties, biotic | Cattle | 2000 | 1 |
| Carvalho *et al.* 2018 | 1 | Dung removal | Human + pig combined | 100 | 1 |
| Amore *et al.* 2018 | 1 | Dung removal | Human | 11 | 1 |
| Nunes *et al.* 2018 | 3 | Dung removal, soil bioturbation, seed dispersal | Human + pig combined | 100 | 1 |
| Manning & Cutler 2018 | 1 | Dung removal | Cattle | 550 | 1 |
| Santos-Heredia *et al.* 2018 | 4 | Dung removal, soil bioturbation, seed dispersal, seed burial | Howler monkey | 30 | 1 |
| Iida *et al.* 2018 | 1 | Dung removal | Sika deer | Natural | 1 |
| González-Tokman *et al.* 2018 | 1 | Dung removal | Cattle | 1000 | 1 |
| França *et al.* 2018 |  | Dung removal, soil bioturbation | Human + pig combined | 200 | 1 |
| Braga *et al.* 2017 | 1 | Seed dispersal | Human + pig combined | 200 | 1 |
| Frank *et al.* 2017 | 1 | Dung removal | Cattle, horse, sheep, deer, fox, wild boar | 220, 34, 50, 32, 14.5, 47 | 6 |
| Lugon *et al.* 2017 | 1 | Seed dispersal | Tapir, muriqui | Unclear | 2 |
| França *et al.* 2017 | 2 | Dung removal, soil bioturbation | Human + pig combined | 200 | 1 |
| Slade *et al.* 2017 | 5 | Dung removal, plant productivity, GHG, nutrient cycling, microbial activity | Dung (species undetermined) | Unclear | 1 |
| Batilani-Filho & Hernandez 2017 | 1 | Dung removal | Pig, dog | 50 | 2 |
| Piccini *et al.* 2017 | 1 | GHG | Cattle | 300 | 1 |
| Nervo *et al.* 2017 | 3 | Dung removal, Nitrogen content of soil and dung, Plant productivity | Cattle | Unclear | 1 |
| Milotić *et al.* 2017 | 2 | Dung removal, seed dispersal | Cattle, horse, sheep | 200, 300 | 3 |
| Farias & Hernández 2017 | 3 | Organic matter, macro and micronutrients, soil pH | Rotting meat, dung (species undetermined) |  | 1 |
| Ortega-Martínez, Moreno & Escobar 2016 | 1 | Dung removal | Sheep, cattle | 200 | 2 |
| Derhé *et al.* 2016 | 3 | Seed dispersal, dung removal, soil bioturbation | Wallaby | 50 | 1 |
| Slade & Roslin 2016 | 2 | Dung removal, plant productivity | Cattle | 940 | 1 |
| Kenyon *et al.* 2016 | 1 | Dung removal | Kangaroo + wallaby combined | 50 | 2 |
| Johnson *et al.* 2016 | 3 | Water retention in soil, plant productivity, plant nitrogen | Cattle | 800 | 1 |
| Ardali, Tahmasebi & Bonte 2016 | 1 | Seed dispersal | Sheep | Unclear | 1 |
| Santos-Heredia *et al.* 2016 | 2 | Plant nitrogen, Plant phosphorous | Howler monkey | 25 | 1 |
| Menéndez, Webb & Orwin 2016 | 2 | Dung removal, Dung soil carbon cycling | Sheep | 200 | 1 |
| Griffiths *et al.* 2016 | 3 | Seed dispersal, Seedling emergence, seedling recruitment | Human + pig combined | 100 | 1 |
| Manning *et al.* 2016 | 2 | Dung removal, seed dispersal | Cattle | 550 | 1 |
| Slade *et al.* 2016 | 1 | Microbial communities | Cattle | 1200 | 1 |
| Slade *et al.* 2016a | 1 | GHG | Cattle | 1200 | 1 |
| Tixier, Bloor & Lumaret 2015 | 2 | Dung removal, leaf litter removal and decomposition | Dung (species undetermined) | Unclear | 1 |
| Yoshihara & Sato 2015 | 2 | Nutrient cycling, plant productivity | Cattle | 100 | 1 |
| Iwasa, Moki & Takahashi 2015 | 1 | GHG | Cattle | 1000 | 1 |
| Griffiths *et al.* 2015 | 1 | Seed dispersal | Human + pig combined | 100 | 1 |
| Enari & Sakamaki-Enari 2014 | 1 | Seed dispersal | Macaque | Natural | 1 |
| Santos-Heredia & Andresen 2014 | 2 | Seed dispersal, seed movement | Howler monkey | 50 | 1 |
| Nervo *et al.* 2014 | 1 | Dung removal | Dung (species undetermined) | Unclear | 1 |
| Feer *et al.* 2013 | 1 | Seed dispersal | Howler monkey | 80 | 1 |
| Braga *et al.* 2013 | 3 | Dung removal, seed removal, soil bioturbation | Human + pig combined | 70 | 1 |
| Penttilä *et al.* 2013 | 1 | GHG | Cattle | 1200 | 1 |
| Gollan *et al.* 2013 | 1 | Dung removal | Pig | 10 | 1 |
| Dangles, Carpio & Woodward 2012 | 1 | Dung removal | Human, carrion | 40 | 2 |
| Lawson, Mann & Lewis 2012 | 2 | Seedling clustering, seed recruitment | Horse | 280 | 1 |
| Braga *et al.* 2012 | 2 | Dung removal, Fly abundance | Human + pig combined | 70 | 1 |
| Kudavidanage, Qie & Lee 2012 | 1 | Dung removal | Dung (species undetermined) | 75 | 1 |
| Kunz & Krell 2011 | 1 | Seed dispersal | Olive baboon | 30 | 1 |
| Culot *et al.* 2011 | 1 | Seed dispersal | Tamarin | Natural | 1 |
| Giraldo & Escobar 2011 | 4 | Dung removal, soil removal, fly activity, seed removal | Cattle + pig combined | 70 | 1 |
| Slade, Mann & Lewis 2011 | 2 | Dung removal, seed burial | Cattle | 780 | 1 |
| Santos-Heredia, Andresen & Stevenson 2011 | 1 | Seed dispersal | Howler monkey, spider monkey | 10 | 2 |
| Brown *et al.* 2010 | 1 | Soil hydrological properties | Cattle + pig | 2500 | 1 |
| Amézquita & Favila 2010 | 1 | Dung removal | Cattle, spider monkey | 200 | 2 |
| Santos-Heredia, Andresen & Zárate 2010 | 2 | Seed movement, seed survival | Howler monkey, spider monkey | 30 | 2 |
| Lee *et al.* 2009 | 1 | Dung removal | Cattle | 50 | 1 |
| Rosenlew & Roslin 2008 | 1 | Dung removal | Cattle | 1920 | 1 |
| Slade *et al.* 2007 | 2 | Dung removal, seed dispersal | Cattle | 620 | 1 |
| Bang *et al.* 2005 | 3 | Plant productivity, Soil Nitrogen content, soil bioturbation | Cattle | 1000, 500 | 1 |
| Horgan 2005 | 2 | Dung removal, fly activity | Pig | 100 | 1 |
| Bustamante-Sánchez, Grez & Simonetti 2004 | 1 | Dung removal | Cattle | 100 | 1 |
| Chapman, Chapman & Vulinec 2003 | 1 | Seed dispersal | Cattle | 25 | 1 |
| Vulinec 2002 | 1 | Seed dispersal | Dung (species undetermined) | Unclear | 1 |
| Andresen 2001 | 1 | Seed dispersal | Howler monkey | 25 | 1 |
| Feer 1999 | 1 | Seed dispersal | Howler monkey | 30 | 1 |
| Kazuhira, Hideaki & Hirofumi 1991 | 1 | Nitrogen gas loss | Cattle | 100 | 1 |
| Estrada & Coates-Estrada 1991 | 1 | Seed dispersal | Howler monkey | 20 | 1 |

c)


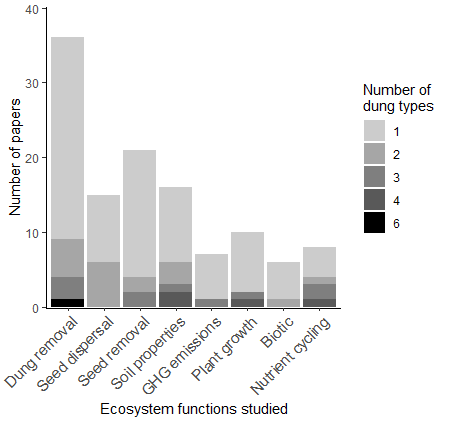

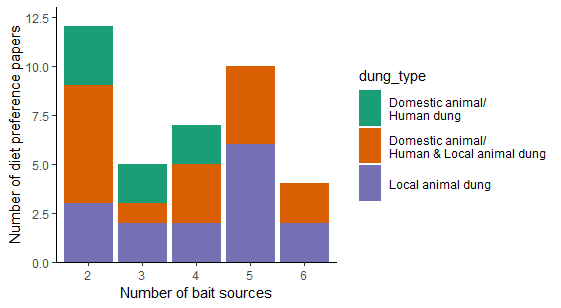

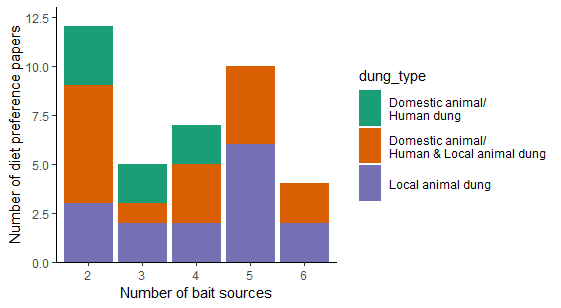

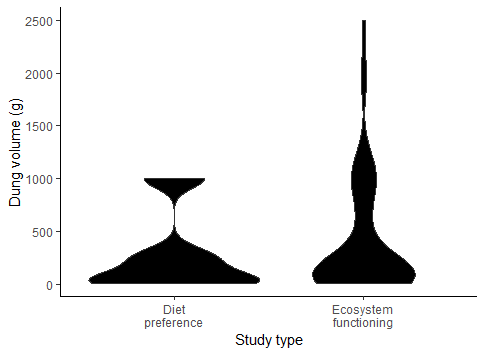

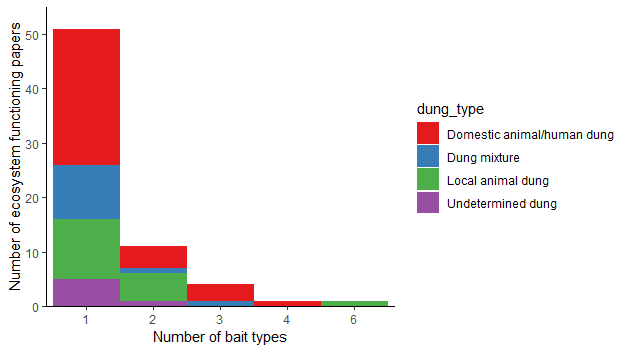

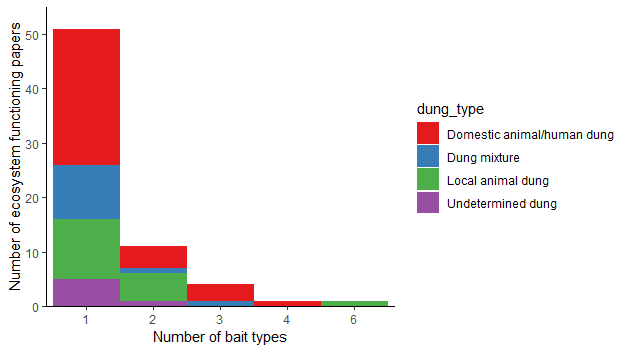


a)

b)

d)

**Figure S1.** Summary of diet preference and ecosystem functioning studies. a) dung volumes used in diet preference and ecosystem functioning studies with frequency of studies represented by polygon size, b) Number of bait sources and dung types used in studies of ecosystem functioning; *Local dung*: dung of mammals that naturally occur in the local area; *Domestic dung/Human dung*: dung of cattle, sheep, pigs, goats, horses (and humans); *Dung mixture*: combination of several dung types to bait one trap, c) studies of ecosystem functions and number of dung types used. *Dung removal*: studies that measured the amount of dung removed from a system by dung beetles; *GHG*: greenhouse gasses released from dung (methane, carbon dioxide, nitrogen gasses); *Plant growth*: plant shoot nitrogen content, plant growth; *Seed removal*: removal of seeds from dung; *Seed dispersal*: horizontal and vertical burial of seeds, seed clustering, seedling recruitment; *Soil properties*: soil characteristics such as bioturbation, nutrient quality; nutrient cycling: soil nitrogen, phosphorous, micro/macro nutrient content; *Biotic*: microbial activity and fly presence, d) diet preference; d) Number of bait sources and dung types used in studies of diet preference; *Local dung*: dung of mammals that naturally occur in the local area; *Domestic dung/Human dung*: dung of cattle, sheep, pigs, goats, horses (and humans).
